# *Chlamydomonas reinhardtii* tubulin-gene disruptants for efficient isolation of strains bearing tubulin mutations

**DOI:** 10.1371/journal.pone.0242694

**Published:** 2020-11-23

**Authors:** Takako Kato-Minoura, Yutaro Ogiwara, Takashi Yamano, Hideya Fukuzawa, Ritsu Kamiya

**Affiliations:** 1 Department of Biological Sciences, Faculty of Science and Engineering, Chuo University, Tokyo, Japan; 2 Biological Science Course, Graduate School of Science and Engineering, Chuo University, Tokyo, Japan; 3 Graduate School of Biostudies, Kyoto University, Kyoto, Japan; University of Illinois at Chicago, UNITED STATES

## Abstract

The single-cell green alga *Chlamydomonas reinhardtii* possesses two α-tubulin genes (*tua1* and *tua2*) and two β-tubulin genes (*tub1* and *tub2*), with the two genes in each pair encoding identical amino acid sequences. Here, we screened an insertional library to establish eight disruptants with defective *tua2*, *tub1*, or *tub2* expression. Most of the disruptants did not exhibit major defects in cell growth, flagellar length, or flagellar regeneration after amputation. Because few tubulin mutants of *C*. *reinhardtii* have been reported to date, we then used our disruptants, together with a *tua1* disruptant obtained from the *Chlamydomonas* Library Project (CLiP), to isolate tubulin-mutants resistant to the anti-tubulin agents propyzamide (pronamide) or oryzalin. As a result of several trials, we obtained 8 strains bearing 7 different α-tubulin mutations and 12 strains bearing 7 different β-tubulin mutations. One of the mutations is at a residue similar to that of a mutation site known to confer drug resistance in human cancer cells. Some strains had the same amino acid substitutions as those reported previously in *C*. *reinhardtii*; however, the mutants with single tubulin genes showed slightly stronger drug-resistance than the previous mutants that express the mutated tubulin in addition to the wild-type tubulin. Such increased drug-resistance may have facilitated sensitive detection of tubulin mutation. Single-tubulin-gene disruptants are thus an efficient background of generating tubulin mutants for the study of the structure–function relationship of tubulin.

## Introduction

Microtubules are fundamental cytoskeletal filaments that play pivotal roles in eukaryotic cell functions such as cell division, intra-cellular transport, cell shape development, and cilia and flagella assembly. Microtubules are produced by polymerization of α/β-tubulin heterodimers. Most eukaryotic cells possess multiple genes encoding α- and β-tubulin. For example, humans possess seven genes that encode α-tubulin and eight genes that encode β-tubulin, with each gene encoding a slightly different amino acid sequence. The presence of multiple genes for the two types of tubulin makes it difficult to study the properties of a particular tubulin species by genetic analysis, because the effects arising from mutation of one of the genes can be masked by the expression of the remaining intact genes.

The single-cell green alga *Chlamydomonas reinhardtii* is a useful experimental organism for studying tubulin function because it possesses a small number of tubulin genes and it produces microtubule-based organelles, flagella. In addition, there is a wide range of genetic tools available and a large amount of biological data has been accumulated for this species. In contrast to the majority of eukaryotes, *C*. *reinhardtii* possesses only two genes (*tua1* and *tua2*) encoding α-tubulin and two genes (*tub1* and *tub2*) encoding β-tubulin [1, 2]. The two genes for each type of tubulin encode the same amino acid sequence [2, 3], and the expression of all four genes is up-regulated after flagellar excision [4]. Whether the two genes in each pair are expressed independently of each other has not yet been firmly established, but the genes do appear to be similarly expressed during flagella formation [4].

Although *C*. *reinhardtii* possess only two genes for each tubulin, the presence of more than one gene expressing the same protein still makes it difficult to isolate tubulin mutants. To date, only five tubulin mutations have been reported: a *tua1* mutation (Y24H) that confers amiprophos-methyl (APM) and oryzalin resistance (upA12) [3]; two kinds of mutations in *tua2* (D205N and A208T) that confer colchicine hypersensitivity (*tua2-1* etc., suppressors of *uni-3-1*, a mutant lacking δ-tubulin) [5]; and two mutations in *tub2* (K350E and K350M) that confer colchicine resistance (*col*^*R*^*4* and *col*^*R*^*15*) [6]. APM, oryzalin, colchicine, and propyzamide (also known as pronamide) are compounds that inhibit tubulin polymerization. These compounds other than colchicine inhibit plant tubulin polymerization at low concentrations and are used as herbicides.

Here, we isolated eight *tua2*, *tub1*, or *tub2* disruptants from an insertional library comprising around 8000 clones prepared based on ref [7]. We also obtained a *tua1* disruptant from the *Chlamydomonas* Library Project (CLiP) [8]. We then used one of the *tub2* disruptants and two double-disruptants possessing only one α-tubulin gene and one β-tubulin gene as parent strains for the production of 20 mutants showing various degrees of resistance to propyzamide and oryzalin. Thus, the use of single-tubulin-gene *C*. *reinhardtii* disruptants enabled efficient isolation of a large number of tubulin mutants resistant to anti-tubulin agents. An early version of this paper has been published as a preprint in bioRxiv (doi: https://doi.org/10.1101/2020.04.07.031005). However, after its publication, 12 of the 32 isolated mutants were accidentally lost. The present paper reports only about the surviving 20 strains.

## Materials and methods

### Isolation of tubulin-gene disruptants

A library of *Chlamydomonas* mutants was prepared by inserting the *aphVIII* gene (paromomycin resistance gene) into the genome [7]. In total, eight tubulin gene disruptants were obtained from this library. Six disruptants, *tua2-B*, *tua2-C*, *tub1-B*, *tub2-A*, *tub2-B*, and *tub2-C* were isolated by performing PCR on the pooled transformants using primers targeting the *aphVIII* sequence (PSI103-F2 and RB02) and two tubulin consensus sequences (3'-Tus1891g and 3'-Tus1803g). A disruptant, *tub1-A*, was isolated using two alternative tubulin consensus primers (5'-Tus1082c and 5'-Tus1596g). A disruptant, *tua2-A*, was isolated using RB02 and an alternative consensus primer (3'-TuA2-3254g). S1 File shows the primers used in the present study. After screening, the disruptants were sequenced in the vicinity of their disrupted tubulin gene (Macrogen Japan Co., Japan).

In addition, a *tua1* disruptant (LMJ.RY0402.158052; referred to as *tua1-A* in the present study) was obtained from the *Chlamydomonas* Library Project [8]; this disruptant has a long insertion composed of two facing paromomycin-resistant CIB1cassettes immediately before the stop codon in *tua1* [8].

The disruptants were backcrossed with wild-type *C*. *reinhardtii* (CC-125) and selected for tubulin-gene disruption by PCR before use. Double disruptants were constructed by standard methods [9], and selected from tetrads by PCR analysis.

### Semi-quantitative real-time PCR

For disruptants *tua1-A*, *tua2-A*, *tub1-B*, and *tub2-A*, semi-quantitative real-time PCR was performed by using TB Green *Premix Ex Taq* GC (Perfect Real Time) and a Thermal Cycler Dice Real-Time System II (Takara, Japan) in accordance with the manufacturer’s instructions.

### Assessment of tubulin protein level and flagellar length during reflagellation

Cells were grown in Tris–acetate–phosphate (TAP) medium [10] under a 12-h light/12-h dark cycle. Deflagellation was induced by pH shock [11]. A second deflagellation was carried out 2 h after the first deflagellation. Before the first deflagellation and after each deflagellation, whole-cell lysates were prepared by following the methods of Wakabayashi et al. [12]. Samples were electrophoresed on 5–20% acrylamide gradient gel [13] and stained with silver [14]. To assess reflagellation activity, aliquots of cells were fixed with formaldehyde/glutaraldehyde solution, and video-recorded under a dark-field microscope. Their flagellar lengths were measured using ImageJ software [15]. More than 50 flagella were measured for each sample. The percentage of cells that had two flagella was 85–100% in pre-deflagellation, 81–100% 2 h after the first deflagellation, and 70–97% 2 h after the second deflagellation.

### Isolation of anti-tubulin drug resistant mutants

Propyzamide Reference Material, Oryzalin Standard, and colchicine were obtained from Fujifilm Wako Pure Chemical Co., Japan. The strains *tub2-A*, *tua1-A* x *tub1-B* (double disruptant) or *tua2-A* x *tub1-B* (double disruptant) were grown to the mid-log phase and then irradiated by ultraviolet light until about 50% of the cells were killed. The culture was spread on TAP/agar plates containing 20 μM propyzamide or 10 μM oryzalin, kept in the dark for 12 h, and then incubated under light for 5–10 days. Colonies that appeared were transferred to liquid medium in 96-well plates containing the same concentration of propyzamide or oryzalin. From each culture that grew, genomic DNA was extracted and subjected to PCR using the following primers: 5'-ChlaTuA1_long969 and 3'-TuA6260 (for *tua1*), 5'-Tua2-10g and 3'-TuA2-3288g (for *tua2*), 5'-tub1-33c and 3'-tub1-1667c (for *tub1*), and 5'-EcoTuB2-upper and 3'-XhoTuB2-lower (for *tub2*). The PCR products were processed for DNA sequencing (Macrogen Japan Co.).

### Drug-resistance test

*C*. *reinhardtii* strains were grown in liquid medium until the mid-log phase, and then diluted to 5 × 10^4^ cells/mL. Then, 30 μL of culture was mixed with 270 μL of growth medium containing propyzamide (0–40 μM) or oryzalin (0–20 μM) in each well of 96-well plates, and cultured for 8 days at 26°C under 12-h light/12-h dark conditions. As a measure of cell proliferation, absorbance at 595 nm was measured 3 h after light onset every day. A wild-type strain (CC-124), an oryzalin-resistant mutant (upA12) [3], or colchicine-resistant mutants (*col*^*R*^*4* and *col*^*R*^*15*) [6, 16] were used as references. This assay was not applicable to the evaluation of colchicine resistance, as TAP media containing colchicine (> 1 mM) often became turbid during culture. The optical density of cells in liquid cultures as well as on the agar plates allowed only qualitative or semi-quantitative assessment of cell proliferation. This is because, in the presence of a high concentration of anti-tubulin drugs, some cells became huge in size while a small fraction of cells assumed an almost normal size [16]. Possibly because of such anomalous growth, some mutants showed slightly higher optical density at higher drug concentrations. Even so, assessment by optical density measurements allowed us to reproducibly assess the drug-resistance of each isolate. The criteria for assessment scores are as follows. For propyzamide: ++, a strain that grew at 40 μM; +, a strain that grew at 4 μM but not 40 μM; +/-, a strain that grew at 2 μM but not > 4 μM (the level exhibited by parental strains); -, a strain that did not grow at 2 μM. For oryzalin: ++, a strain that grew at 20 μM; +, a strain that grew at 5 and10 μM but not at 20 μM; +/-, a strain that did not grow at 5 μM (the level exhibited by parental strains).

As an alternative assessment of cell growth, an equal number of cells from each strain was spotted in a dilution series on agar plates containing different concentrations of propyzamide, oryzalin and colchicine and cultured under the same conditions as above. In this case, the effect of each drug was visually assessed from the density of cells on the plate after 1 week of culture. The two methods yielded qualitatively similar results. The colchicine resistance given in Table 1 was based on the following criteria. +: a strain whose density cultured at 4 mM colchicine was similar to that of its parental strain cultured without colchicine, and much higher than that of the parental strain cultured at 4 mM colchicine. +/-: a strain whose density at 2 mM colchicine was similar to that of the parental strains cultured without colchicine, but much lower than the latter when cultured at 4 mM colchicine. -: a strain whose density was similar to that of the parental strain cultured without colchicine but lower when cultured at 1–4 mM colchicine.

**Table 1:** Tubulin missense mutants isolated in this study.

Strain	Parent strain	Gene altered	Mutation (DNA)	Mutation (protein)	Growth	Resistance to		
						propyzamide	oryzalin	colchicine^d^
*tub2-A*					normal^c^	+/-	+/-	+/-
*tua1-A* x *tub1-B*					normal^c^	+/-	+/-	+/-
*tua2-A* x *tub1-B*					normal^c^	+/-	+/-	+/-
*ory2*	*tua2-A* x *tub1-B*	*tua1*	TAC->CAC^a1^	Y24H^a1^	normal	-	++	+/-
*ory3*			TTC->CTC	F52L	normal	+	++	+/-
*ory4*			TCC->GCC	S165A	normal	+/-	++	+/-
*ory5*					normal	+/-	++	+/-
*pyz9*	*tub2-A*		TTC->TTA	F351L	normal	+	+/-	-
*ory6*	*tua1-A* x *tub1-B* (E2)	*tua2*	TAC->AAC	Y24N	normal	+/-	+	+/-
*ory7*			TTC->TGC	F49C	normal	+/-	+	+/-
*ory8*			TTC->GTC	F138V	slow	+/-	++	+/-
*pyz10*	*tub2-A*	*tub1*	GAG->AAG	E198K	normal	++	+/-	+/-
*pyz11*					normal	++	+	+/-
*pyz12*			TTC->TGC	F266C	normal	++	+/-	+
*pyz13*					normal	++	+/-	+
*pyz14*					normal	++	+/-	+
*pyz2*	*tua2-A* x *tub1-B*	*tub2*	CAG->CAT	Q134H	normal	+	+/-	+/-
*pyz3*					slow	+	+/-	-
*pyz4*			GAG->AAG	E198K	normal	++	+	+
*pyz5*			ATC->AAT	I236N^b^	normal	++	+	+
*pyz6*			AAG->GAG^a2^	K350E^a2^	slow	++	+	+
*pyz7*			ATC->TTC	I368F	normal	++	+/-	-
*pyz8*					normal	++	+/-	-

++, highly resistant; +, resistant; +/-, parent-strain level; -, hypersensitive. For criteria, see text.

^a1^Mutation previously reported for upA12 [3].

^a2^Mutation previously reported for *col*^*R*^*4* [6].

^b^Mutation found in human cancer cells resistant to 2-methoxyestradiol [36].

^c^Compared with a wild type (CC-124).

^d^Assessed by visual inspection of 7-d cultures on agar plates containing 0–4 mM colchicine.

### Three-dimensional structure prediction of *C*. *reinhardtii* α/β-tubulin heterodimer

The three-dimensional structure of the *C*. *reinhardtii* α/β-tubulin heterodimer was predicted by using FAMS software [17] based on a known tubulin tetramer structure obtained from the Protein Data Bank (PDB ID: 1Z2B [18]). To determine the amino acids that most likely interacted with the examined drugs, *in silico* molecular docking analyses were performed using the ChooseLD program [19].

### 2D-PAGE of isolated axonemes

Axonemes were isolated from the *C*. *reinhardtii* strains by using standard procedures [11]. A small aliquot of axonemal precipitate (~2 or 10 μg) was extracted with a buffer containing 5 M urea and 2 M thiourea and analyzed by 2D-PAGE as described previously [20]. Since α- and β-tubulin are modified post-translationally, the loading amount was adjusted so that their major forms only were detectable by silver staining. The predicted pI values of the wild-type and mutant tubulins were calculated by using the EMBOSS database and the Sequence Manipulation Suite, which is a collection of JavaScript programs for examining short protein sequences (https://www.bioinformatics.org/sms2/protein_iep.html) [21].

## Results

### Isolation of tubulin-gene disruptants

**Fig 1:**
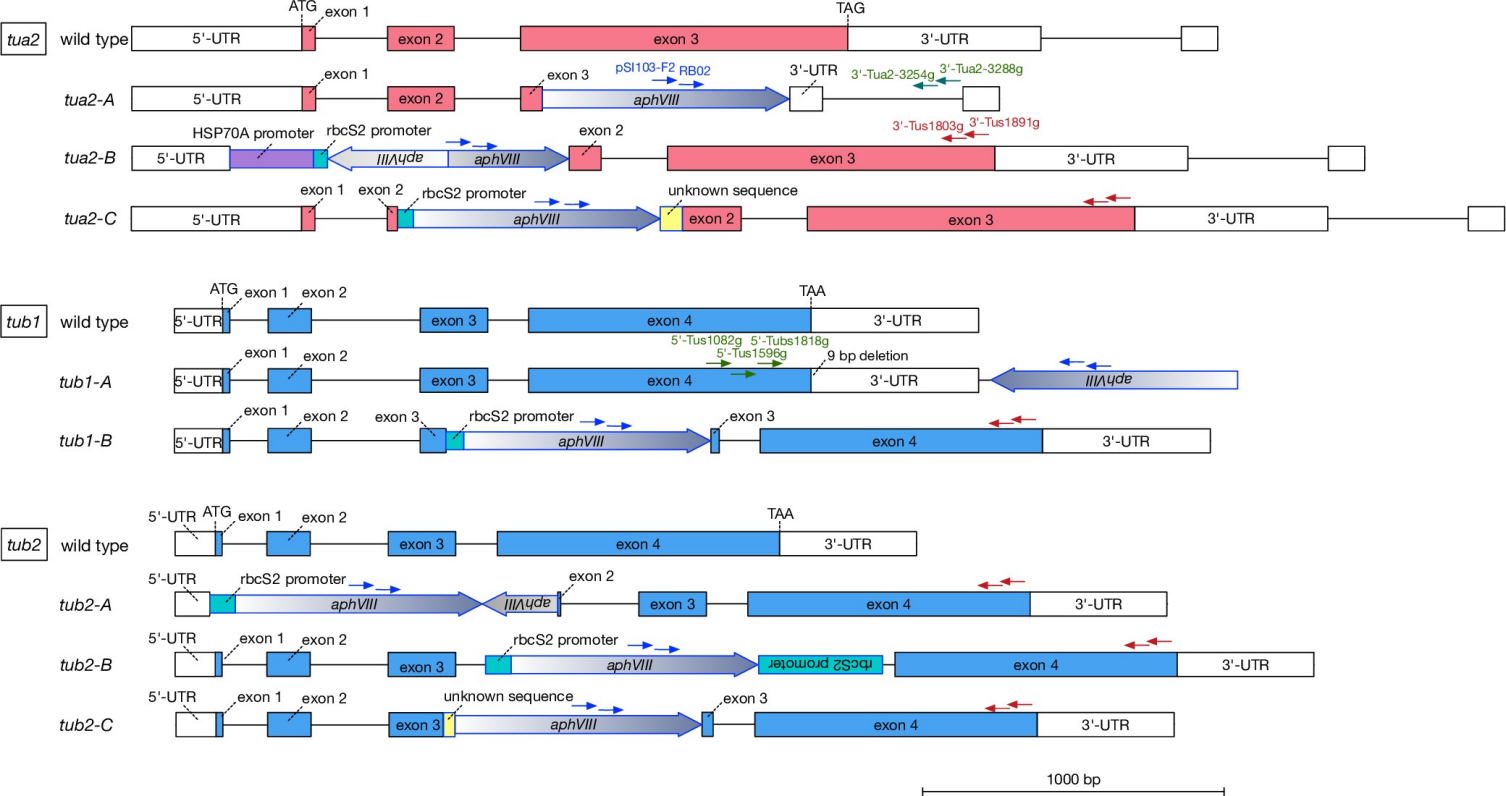
Structures of the *tua2*, *tub1*, and *tub2* genes in wild type and the eight tubulin-gene disruptants. Structures of the tubulin genes in wild type and disruptants. The *aphVIII* insertion sites in the disrupted genes are shown as large gray arrows. Exons are shown as boxes and introns as lines between boxes. Start and stop codons are indicated in the diagrams of the wild-type genes. Arrows show the primers used for screening. Blue arrows indicate primers targeting the *aphVIII* sequence (PSI103-F2 and RB02). Red arrows indicate tubulin consensus sequences (3'-Tus1891g and 3'-Tus1803g). Green arrows with labels indicate other tubulin-specific primers used for detecting gene disruption in some strains. Primers and their sequences are listed in S1 File.

**Fig 2:**
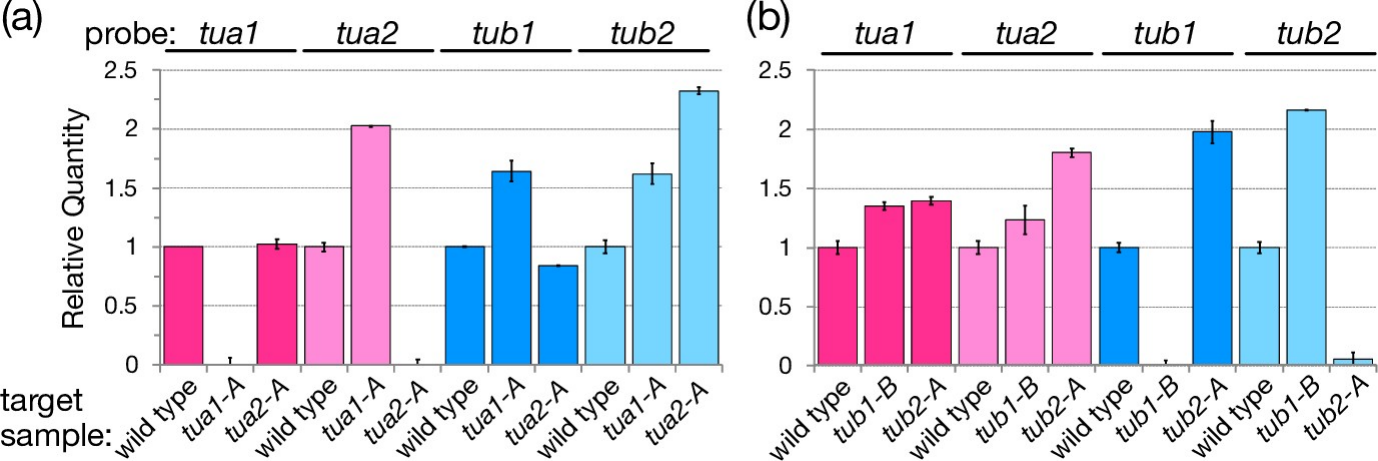
Semi-quantitative real-time polymerase chain reaction in the tubulin-gene disruptants. The transcription levels of the four tubulin genes were analyzed by using total RNA extracted from (a) wild type (CC-125) and two α-tubulin gene disruptants (*tua1-A* and *tua2-A*), and (b) from wild type and two β-tubulin gene disruptants (*tub1-B* and *tub2-A*). Data are means ± SD (n = 3).

A library of around 8000 clones was prepared by *aphVIII* gene cassette insertion into the genome according to the methods reported previously [7]. The library was then screened by PCR using primer pairs consisting of one primer targeting a consensus sequence of the four tubulin genes and another primer targeting the *aphVIII* fragment. As a result, we isolated eight tubulin gene disruptants: three showing *tua2* disruption (*tua2-A*, *tua2-B*, *tua2-C*), two showing *tub1* disruption (*tub1-A*, *tub1-B*), and three showing *tub2* disruption (*tub2-A*, *tub2-B*, *tub2-C*). Fig 1 shows the sites of the *aphVIII* cassette insertion in the eight disruptants, and S1 Fig shows the PCR confirmation of the structure of the disrupted genes. In six of the disruptants (*tua2-A*, *tua2-B*, *tua2-C*, *tub1-B*, *tub2-A*, *tub2-C*), the *aphVIII* cassette was inserted into the gene. In the remaining two disruptants, the *aphVIII* cassette was inserted after the open reading frame (*tub1-A*) or within an intron (*tub2-B*). In all eight disruptants, *AphVIII* cassette insertion resulted in complete absence of mRNA expression of the affected tubulin gene, as confirmed by northern blot analysis (S2 Fig). For *tua1-A*, *tua2-A*, *tub1-B*, and *tub2-A*, semi-quantitative real-time PCR was performed and again no expression of mRNA from the tubulin genes was detected (Fig 2).

**Fig 3:**
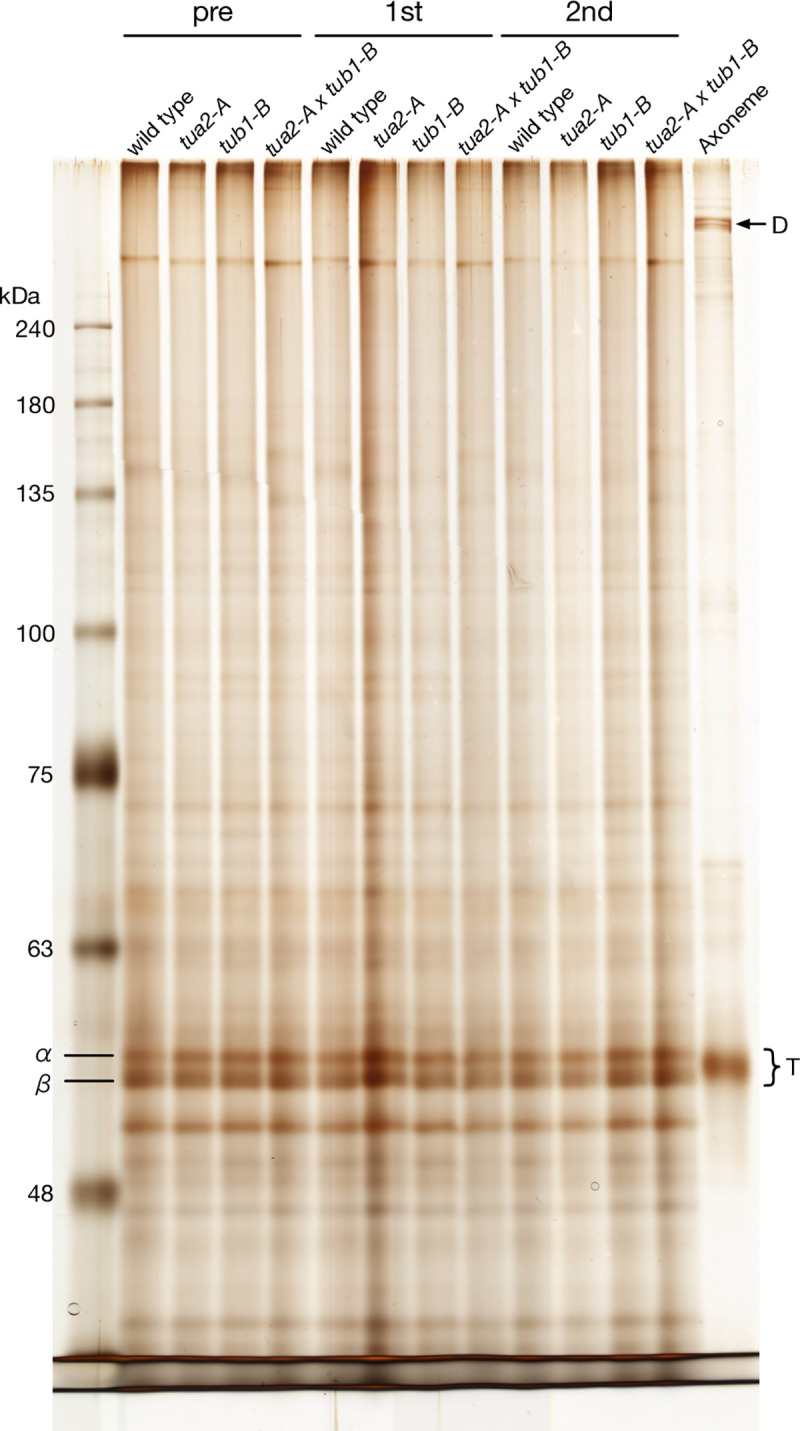
Tubulin protein expression in the tubulin-gene disruptants. All of the disruptants showed tubulin expression at the normal level. Whole-cell lysates from wild type (CC-125), *tua2-A*, *tub1-B*, and a *tua2-A* x *tub1-B* double mutant were prepared before deflagellation (pre), after the 1st deflagellation (1st), and after the 2nd deflagellation (2nd) and electrophoresed. Wild-type axoneme (Axoneme) was loaded for comparison. D indicates bands with axonemal dynein heavy chains (~500 kDa). T indicates α and β-tubulin.

**Fig 4:**
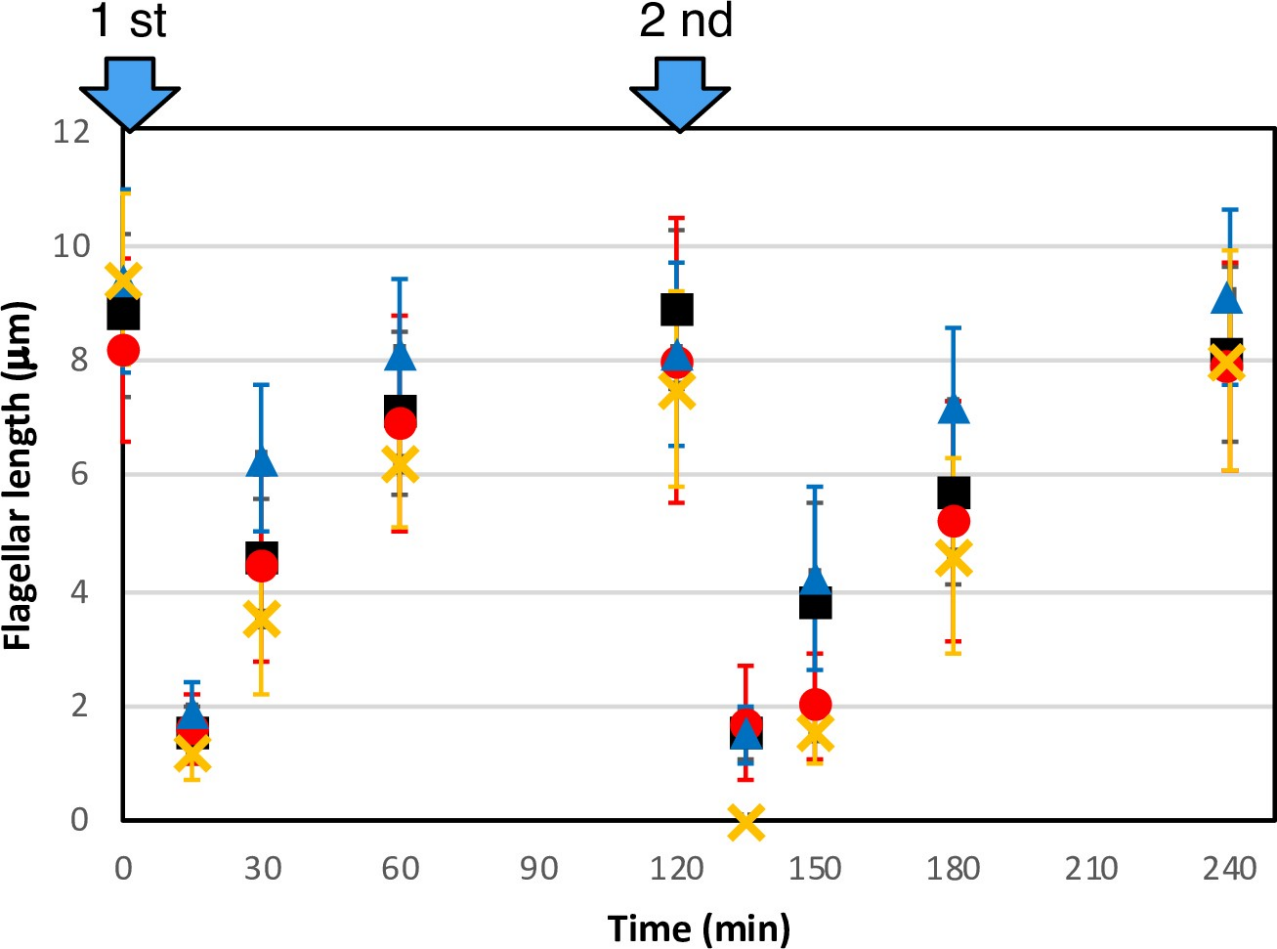
Change in flagellar length after repeated deflagellation in the tubulin-gene disruptants. Horizontal axis indicates time after the 1st deflagellation. Arrows indicate the timing of the 1st and 2nd deflagellation by acid treatment followed by neutralization. Black squares, wild type (CC-125); red circles; *tua2-A*, blue triangles; *tub1-B*; yellow crosses; a *tua2-A* x *tub1-B* double mutant. Error bars: SDs (n = 50).

The growth rate of the eight disruptants was reduced to varying degrees; while five disruptants grew at almost the wild-type rate, *tua2-B* and *tub1-A* displayed very slow growth rates and *tub2-B* displayed intermediate rates. These slow-growing strains were not used for subsequent studies. The two double disruptants used as parent strains for isolation of drug-resistant mutants grew at almost the normal rate (S1 Table, S3 Fig). For *tua2-A*, *tub1-B*, and the double disruptant *tua2-A*×*tub1-B*, tubulin expression (Fig 3), and the time course of flagellar regeneration after amputation (Fig 4) were normal, suggesting that the disruptants still produced sufficient α/β-tubulin heterodimer for their cellular functions via the remaining intact genes. The mean flagellar length was comparable among the disruptants (see Fig 4). The tubulin disruptants showed some difference in their sensitivity to anti-tubulin drugs, although the sensitivity somewhat varied among alleles (S1 Table, S3 Fig). Therefore, the tubulin missense mutants obtained in this study, as shown below, were examined for their drug sensitivity relative to the sensitivity of each parental strain.

### Mutant isolation using tubulin-gene disruptants

We used the disruptants to isolate *C*. *reinhardtii* strains expressing tubulins with missense mutations that confers resistance to propyzamide or oryzalin. Three parent strains were used: the single disruptant *tub2-A*, a double disruptant generated by crossing *tua1-A* with *tub1-B*, and a double disruptant generated by crossing *tua2-A* and *tub1-B*. As detailed in Materials and Methods, these strains were first mutagenized by UV and inoculated on drug-containing agar plates, and colonies that appeared were cultured in drug-containing liquid media. The clones that grew were saved as drug-resistant mutants, and the sequence of their tubulin genes were determined.

As a result of 1–3 trials with each parental strain against oryzalin or propyzamide, 20 strains showing a total of 14 different tubulin missense mutations were isolated. Mutants first selected for oryzalin-resistance were given strain names with *ory*, while those selected for propyzamide-resistance were given names with *pyz*. Specifically, PCR and DNA sequencing identified 1 *tua* and 5 *tub* mutants in 8 isolates from the *tub2-A* disruptant, 3 *tua* and 8 *tub* mutants in 22 isolates from the *tua1-A*×*tub1-B* double disruptant, and 4 *tua* mutants and 11 *tub* mutants in 23 isolates from the *tua2-A*×*tub1-B* double disruptant. The overall probability of an isolate having a mutation in one of the tubulin genes was ~60% (however, 12 of the 32 isolated were accidentally lost). We assessed the sensitivity of each isolate to propyzamide and oryzalin by measuring the culture's density at 595 nm every 24 h in the presence of these drugs (S2 Table). At the same time, each isolate was cultured on agar plates containing various concentrations of these drugs and colchicine, and the density of cells after 1 week of culture was regarded as a measure of resistance (S4 Fig).

Table 1 shows the obtained mutants classified by the gene affected, as well as the results of a semi-quantitative assessment of each strain’s resistance to oryzalin and propyzamide. Most of the *ory* strains and a *pyz* strain (*pyz9*) had a missense mutation in an α-tubulin gene. In contrast, most of the *pyz* strains, other than *pyz9*, had mutations in a β-tubulin gene.

**Fig 5:**
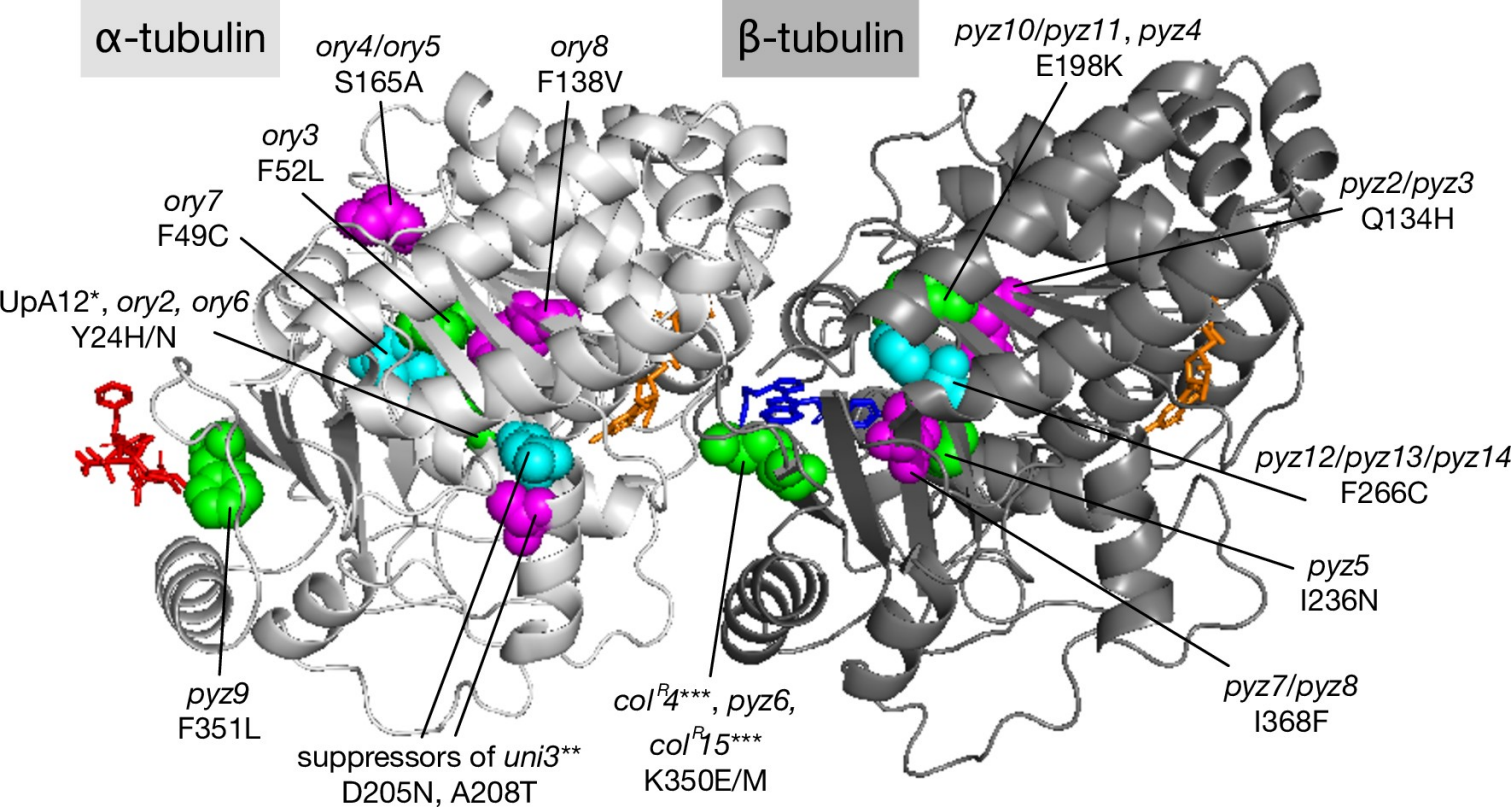
Predicted three-dimensional structure of *Chlamydomonas reinhardtii* α/β-tubulin heterodimer showing the mutations reported in the present and previous studies. Light gray, α-tubulin; dark gray, β-tubulin. Altered amino acids are shown as sphere representations. The binding sites of tubulysin M (red, an oryzalin-like compound) and 2RR (blue, 3-[(4-{1-[2-(4-aminophenyl)-2-oxoethyl]-1H-benzimidazol-2-yl}-1,2,5-oxadiazol-3-yl)amino]propanenitrile, a propyzamide-like compound) were determined by applying the alignment command in MacPyMol software to the tubulin structures 4ZOL and 4O2A reported in the presence of these compounds [25, 26]. The orange stick representations show GTP (in α-tubulin) and GDP (in β-tubulin). Mutations identical to previously reported mutations are marked with asterisks: *, [3]; **, [5]; and ***, [6].

Fig 5 shows a predicted three-dimensional structure of *C*. *reinhardtii* α/β-tubulin heterodimer labeled with the site of each missense mutation reported here and in previous studies [3, 5, 6]. Two of the isolates had mutations that have been reported previously: *ory2* had a *tua1* Y24H mutation as did upA12 [3]; *pyz6* had a *tub2* K350E mutation as did *col*^*R*^*4* [6]. Interestingly these mutants displayed a slightly stronger drug resistance than the previously-reported corresponding mutants; *ory2* exhibited a stronger oryzalin-resistance than upA12, and *pyz6* exhibited a stronger propyzamide-resistance than *col*^*R*^*4* (S4 Fig). Their stronger drug-resistances possibly reflects the fact that the mutants isolated here express only mutated tubulin from a single gene, whereas the previously-reported mutants express a mutated tubulin together with a wild-type counterpart.

**Fig 6:**
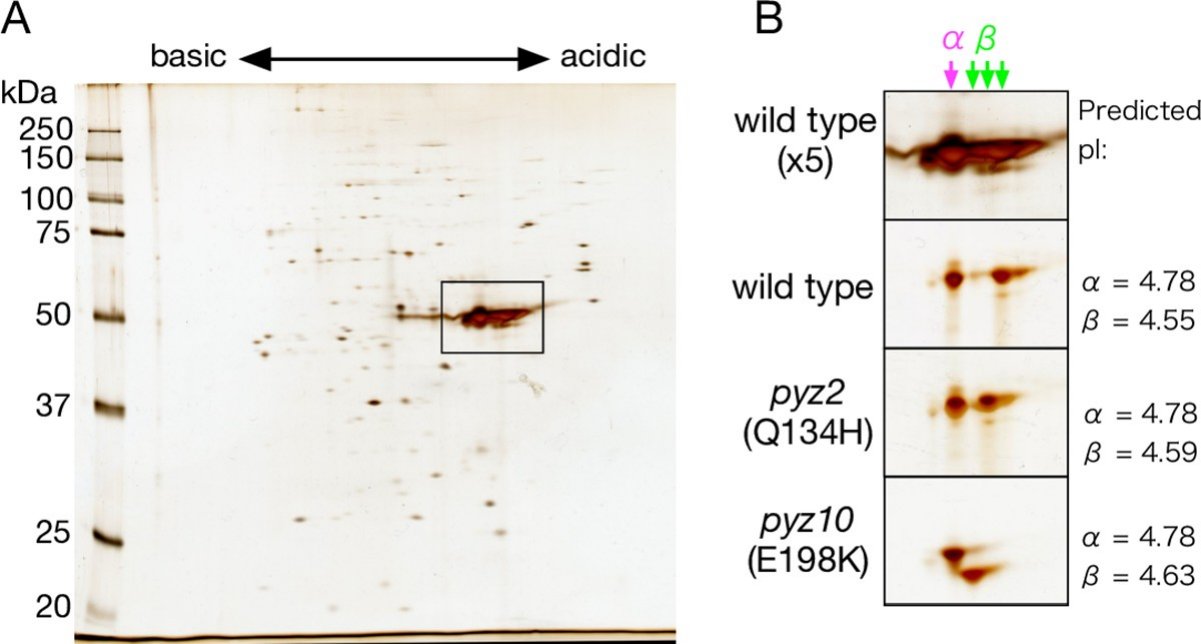
Two-dimensional polyacrylamide gel electrophoresis analysis of axonemes from strains *pyz2* and *pyz10*. Protein extracts of axonemes of wild type (CC-125), *pyz2* and *pyz10* were loaded on a two-dimensional polyacrylamide gel and stained with silver. pH range: 4.0–7.0. (A) Electrophoresis pattern of wild-type axoneme (~10 μg loaded). (B) Portions of polyacrylamide gels showing the major spots of α- and β-tubulin. Upper panel shows a close-up of the area indicated by the box in (A). The lower three panels show the polyacrylamide gels after loading approximately 2 μg of axoneme. The predicted pIs of the wild-type and three mutant α- and β-tubulins are indicated to the right of the panels.

Some of the identified mutations involved the substitution of amino acids with different charges. For example, *pyz2*/*pyz3* (we use a slash to separate strains that have the same tubulin genotypes) expressed β-tubulin with the mutation Q134H and *pyz4* and *pyz10*/*pyz11* expressed β-tubulin with the mutation E198K. The isoelectric point (pI) values of these β-tubulins predicted from their amino acid sequences were 4.59 and 4.63, respectively, which were greater than the pI of wild-type β-tubulin (4.55). We confirmed the expression of β-tubulins with different pIs in those strains by two-dimensional polyacrylamide gel electrophoresis (2D-PAGE) of axonemal proteins from the mutants and wild type (Fig 6). As expected, the spot of β-tubulin appeared at higher pH values in the order *pyz10* (E198K) > *pyz2* (Q134H) > wild type. The 2D-PAGE analysis also verified that each mutant expressed β-tubulin from only a single gene, since it detected no β-tubulin spots with the wild-type pI in mutant samples.

### Novel tubulin mutant strains exhibited various sensitivities to anti-tubulin agents

**Fig 7:**
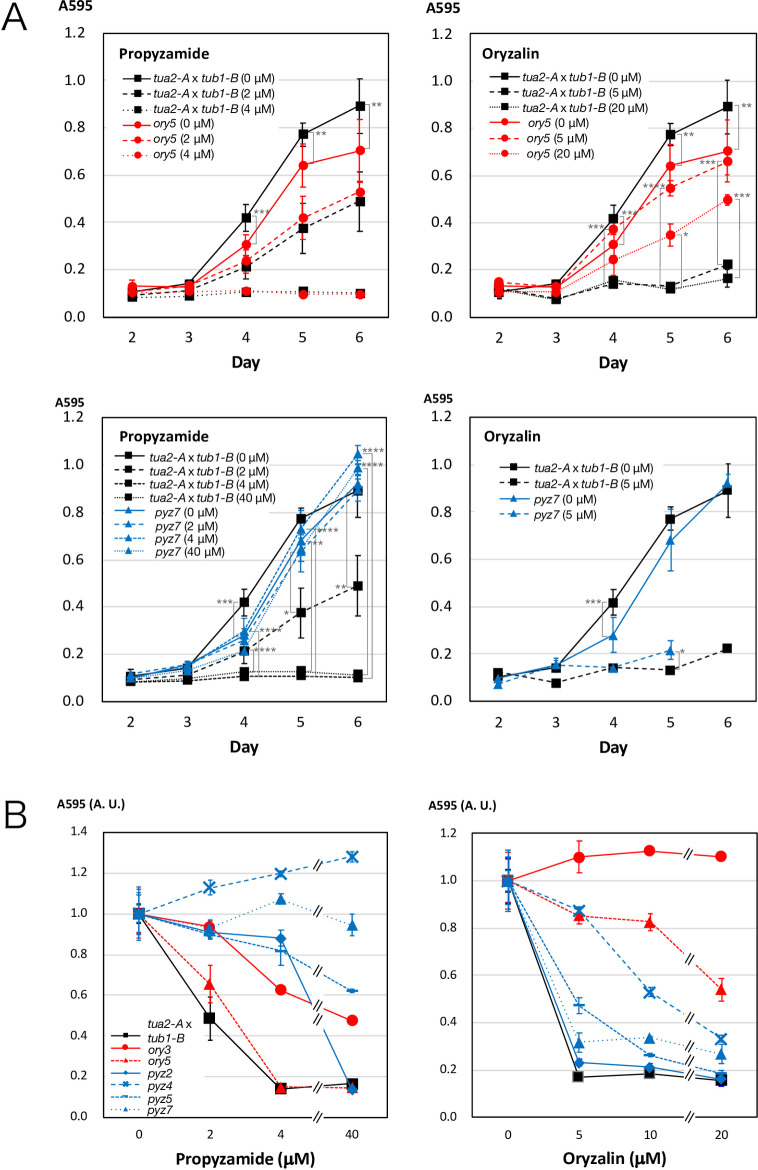
Growth profiles of representative missense mutants in the presence of propyzamide and oryzalin. Cells were cultured in growth medium containing different concentrations of propyzamide and oryzalin. Absorbance of the culture was measured at 595 nm every day. Error bars represent standard deviations in three independent measurements. (A) Growth curves of two representative strains, *ory5* and *pyz7*, compared with the parent strain *tua2-A* x *tub1-B* at two or three concentrations of drugs. *ory5* is resistant to oryzalin but not to propyzamide. In contrast, *pyz7* is resistant to propyzamide but not to oryzalin. *, *P*<0.05; **, *P*<0.01; ***, *P*<0.0001; ****, *P*<0.00001 (ANOVA test). (B) Relative optical densities 5 days after the initiation of culture are plotted against the concentration of propyzamide or oryzalin. Seven strains, *ory3*, *ory5*, *pyz2*, *pyz4*, *pyz5*, *pyz7*, and the parent strain *tua2-A* x *tub1-B* are compared. Optical densities at 0 concentration are normalized to 1.

The mutant strains obtained, most of which are novel in *Chlamydomonas*, displayed various patterns of sensitivity to anti-tubulin agents (Table 1 and Fig 7). Most of the mutants selected in oryzalin-containing media, *ory2*, *ory3*, *ory4*, *ory5*, and *ory8*, naturally showed high oryzalin resistance and grew well in the presence of 20 μM oryzalin. Of these, *ory2* showed hypersensitivity to propyzamide. In contrast, *ory3* was slightly resistant to propyzamide. Likewise, ten strains of mutants first selected on propyzamide-containing media, *pyz4*, *pyz5*, *pyz6*, *pyz7*, *pyz8*, *pyz10*, *pyz11*, *pyz12*, *pyz13*, and *pyz14*, showed particularly strong propyzamide resistance and grew well in growth medium containing 40 μM propyzamide, where parental strains (*tub2-A*, *tua1-A*✕t*ub1-B*, *tua2-A*✕ *tub1-B*) were barely viable (Fig 7, S2 Table). Many *pyz* strains (those with the mutation F266C, I236N, or K350E) exhibited strong resistance to colchicine. Such cross-resistance has been observed in K350E [16]. In contrast, strains having Q134H or I368F mutation displayed no resistance despite their strong resistance to propyzamide (S4 Fig). The different sensitivities to colchicine and propyzamide in these mutants are interesting because the two agents bind to almost the same position on the tubulin heterodimer [22].

## Discussion

By screening an *AphVIII* insertional library, we isolated three disruptants lacking *tua2*, two lacking *tub1*, and three lacking *tub2*. All were most likely null mutants (S2 Fig). Although these disruptants lacked one of their tubulin-encoding genes, their cytoplasmic tubulin levels remained normal (Fig 3), suggesting the presence of an auto-regulatory mechanism that maintains the tubulin mRNA level, as observed in other eukaryotic cells [23]. Indeed, in *tua1-A*, *tub1-B*, and *tub2-A*, the mRNA expression level of the remaining α- or β-tubulin gene was increased approximately 2-fold compared with wild type (Fig 2). Also, flagellar length, ability to produce flagella after amputation (Fig 4), and in most of the disruptants, overall cell growth rate did not greatly differ from the wild-type growth rate (S3 Fig). Thus, although *C*. *reinhardtii* possesses two α-tubulin genes and two β-tubulin genes, a single gene for each type is enough to supply the tubulin necessary for its cellular functions. However, the present findings do not mean that the two genes for each tubulin have exactly the same function; rather, the two genes may differ from each other in a subtle manner. For example, the sensitivity to anti-tubulin drugs varied among the disruptants (S3 Fig). Although it could be due to some non-tubulin mutation(s) unintentionally introduced in these disruptants, there may be some difference in the regulation of gene expression that is dependent on the concentration of free tubulin in the cytoplasm [24]. Thus, how the two genes encoding the two tubulins differ in their function and regulation warrants further investigation, and our single-tubulin-gene mutants established here should be useful for such investigations.

Next, we used the disruptants to obtain mutants with resistance to two anti-tubulin agents, propyzamide and oryzalin. Several rounds of trial to isolate mutants resistant to one or both of the agents afforded 8 mutants with 7 different α-tubulin gene missense mutations and 12 mutants with 7 different β-tubulin gene missense mutations (Table 1). The number of mutations obtained is much larger than the total number that has been reported previously (i.e., 3 α-tubulin mutations and 2 β-tubulin mutations) [3, 5, 6]. In addition, we found that about 60% of the clones isolated on the basis of drug resistance harbored a mutation in a tubulin gene. Together, these findings suggest that our approach of using single-tubulin-gene disruptants is a highly efficient means of obtaining tubulin mutant strains. The high efficiency may be partly due to the stronger effect of tubulin mutations in single-tubulin-gene strains than in ordinary strains (S4 Fig, S2 Table), and partly due to the easier detection of tubulin mutations in strains with fewer tubulin genes.

How the sensitivity to anti-tubulin agents varied in the mutants is an important issue that warrants clarification. Some of the tubulin mutants that conferred resistance to the anti-tubulin agents had a mutation near to where the anti-tubulin agents bind to the α/β-tubulin heterodimer (Fig 5). The binding site of oryzalin, inferred from that of an analogous compound, tubulysin M, is at the intra-dimer interface [25] close to the α-tubulin mutation F351L (in strain *pyz9*). Other *ory* mutants whose mutation sites occur independently of the tubulysin M-binding site may confer oryzalin resistance by modulating the three-dimensional structure of α-tubulin. Likewise, a propyzamide-like compound, 2RR, is known to bind at the inter-dimer interface [26] close to several of the identified β-tubulin mutation sites: E198K (in *pyz4* and *pyz10*/*pyz11*), I236N (in *pyz5*), K350E (in *col*^*R*^*4* and *pyz6*), and I368F (in *pyz7*/*pyz8*). Other mutations may confer propyzamide resistance through some structural change in α/β-tubulin.

Several of the mutations detected in the present study are similar to those reported in other organisms (S3 Table). For α-tubulin, mutation F49C in *ory7*, F52L in *ory3*, and S165A in *ory4*/*ory5* are analogous to the mutations reported in a *Toxoplasma gondii* oryzalin-resistant mutants [27, 28]. For β-tubulin, mutation Q134H in *pyz2/pyz3* is analogous to the mutation in a *Beauveria bassiana* benzimidazole-resistant mutant [29]; mutation E198K in *pyz4* and *pyz10*/*pyz11* corresponds to the mutation in fungi and nematodes that confers benzimidazole resistance and phenylcarbamate hypersensitivities [30–35]; I236N in *pyz5* is analogous to the mutation responsible for resistance to the anti-cancer drug 2-methoxyestradiol in human epithelial cancer cells [36]. Mutations Y24N, F138V, and F351L in α-tubulin (*ory6*, *ory8*, and *pyz9*) and mutations F266C and I368F in β-tubulin (*pyz12*/*pyz13*/*pyz14* and *pyz7*/*pyz8*) are being reported here for the first time; further investigations are needed to examine whether mutations analogous to these mutations cause altered drug sensitivity in other organisms.

Although the present study selected mutants based only on their resistance to two anti-tubulin agents, use of other agents such as the microtubule-stabilizing agent taxol, or screening for other properties such as hypersensitivity to drugs, resistance to low temperature, or deficiency in flagellar formation and motility will lead to the isolation of a greater variety of mutants. Detailed analyses of many such mutants will deepen our understanding of the structure–function relation of tubulins. Since one of the mutations identified in the present study corresponded to mutation found in human tubulins that confer drug resistance in cancer cells, we expect that studies of *Chlamydomonas* tubulin mutants will also contribute to the development of improved cancer therapies.

## Supporting information

S1 File (PDF)

S1 Fig Tubulin-gene disruptions confirmed by PCR using primers specific to *AphVIII* and the target tubulin gene. In each disruptant, the structure of the affected tubulin gene was confirmed by PCR using primers specific to *AphVIII* (RB02) and the target tubulin gene. A wild type (CC-125) genomic DNA was used as the template for negative controls. PCR-amplified gene fragments contained the inserted *AphVIII* cassette from the genomic DNA templates extracted from the disruptants but not the wild type. The sizes of the amplified fragments matched those predicted from the manner of *AphVIII* gene insertion (Fig 1), which was determined by sequencing in the vicinity of their disrupted tubulin gene.
(TIF)

S2 Fig Northern blotting to confirm tubulin gene expression in wild-type *Chlamydomonas reinhardtii* and the eight tubulin-gene disruptants. (a) *tua1* transcripts. Total RNA from a wild type (CC-125), *tua1-A*, *tua2-A*, *tua2-B*, and *tua2-C* were hybridized with *tua1* mRNA-specific probes. (b) *tua2* transcripts. Total RNA from *tua2-A*, *tua2-B*, *tua2-C*, and wild type were hybridized with *tua2* mRNA-specific probes. (c) *tub1* transcripts. Total RNA from wild type, *tub1-A*, *tub1-B*, *tub2-A*, *tub2-B*, and *tub2-C* were hybridized with *tub1* mRNA-specific probes. (d) *tub2* transcripts. Total RNA from wild type, *tub1-A*, *tub1-B*, *tub2-A*, *tub2-B*, and *tub2-C* were hybridized with *tub2* mRNA-specific probes.
(TIF)

S3 Fig Growth rate and drug sensitivities of the eight tubulin-gene disruptants. Cells were cultured in growth medium containing different concentrations of anti-tubulin drugs. Absorbance of the culture was measured at 595 nm every day. For comparison, data of a wild type (CC-124) was also collected. Error bars represent standard deviations of three different measurements. A: Growth rate of the tubulin disruptants. B: Drug sensitivities. Relative optical densities on day 5 are shown for all strains except for slow-growing strains, *tua2-B*, *tub1-A*, and *tub2-B*. For the slow-growing strains, data on day 8 are shown. In each case, the optical density observed without a drug is normalized to 1.
(TIF)

S4 Fig Growth of the missense mutants on agar plates containing anti-tubulin drugs. Serial dilutions of the cells were inoculated onto TAP/agar medium containing various concentrations of propyzamide, oryzalin and colchicine, and cultured for 7 days. The names of strains are given in the table (upper right corner) in different colors reflecting their parent strains. Three parent strains and three previously reported strains, upA12, *col*^*R*^*4*, and *col*^*R*^*15*, were also cultured and compared with *ory2* and *pyz6* having the same mutations (boxed). The latter single-tubulin-gene mutants show slightly stronger drug resistance as seen at high drug concentrations.
(TIF)

S1 Table Growth rate and drug sensitivities of the eight tubulin-gene disruptants. Cells were cultured in growth medium containing various concentrations of anti-tubulin drugs. Absorbance of the culture was measured at 595 nm every day. For comparison, data of a wild type (CC-124) were also collected. Standard deviations in three different measurements are also shown. The green background shows a rough measure of cell density for qualitative comparison of growth extent.
(XLSX)

S2 Table Growth rate and drug sensitivities of the tubulin missense mutants. Absorbance at 595 nm was measured as in S1 Table. For comparison, data of parent strains and previously reported mutants, upA12, *col*^*R*^*4*, and *col*^*R*^*15*, were also collected.
(XLSX)

S3 Table Drug-resistant tubulin missense mutants identified in *Chlamydomonas reinhardtii* and other organisms. (XLSX)

S1 Raw images (JPEG)

S2 Raw images (PDF)

S3 Raw images (JPEG)

S4 Raw images (PDF)
