# Supplementary material for: Chlamydomonas reinhardtii tubulin-gene disruptants for efficient isolation of strains bearing tubulin mutations
Source: PLoS One. 2020 Nov 23;15(11):e0242694. doi: 10.1371/journal.pone.0242694 (PMC7682851; doi:10.1371/journal.pone.0242694)
Supplement: S1 File — (PDF) [file pone.0242694.s001.pdf]

Chlamydomonas reinhardtii Tubulin-Gene Disruptants for Efficient Isolation of Strains Bearing Novel Tubulin Mutations

Takako Kato-Minoura[1, 2]*, Yutaro Ogiwara[2], Takashi Yamano[3], Hideya Fukuzawa[3], and Ritsu Kamiya[1]

S1 Appendix. Primers used in this study.

PSI103-F2: 5'-TGTCCGTTCGATCGCAGTCT-3'

RB02: 5'-GTCGACTTGGAGGATCTGGACGA-3'

3'-Tus1891g: 5'-GCCVKCSYCCTCGGCGGA-3'

3'-Tus1803g: 5'-ACCCTCSTCCATRCCCTCACCGA-3'

5'-Tus1082c: 5'-GGCCGCATGTCGACCAAGGAGGT-3'

5'-Tus1596g: 5'-GTCGTACTTCGTCGAGTGG-3'

3'-TuA2-3254g: 5'-CCAATCTCATTCATGCACTTCCTGTTTGTC-3'

5'-Tub1-3175c: 5'-CGGCTATTTAGGCGAGCGGTCTA-3'

3'-Tub1-3683g: 5'-CTTATGGGATTGGTTCAGCGTTAAC-3'

5'-Tub2-3032c: 5'-CGTGACTGTGGCGGCCTTG-3'

3' -Tub2-3507g: 5'-GACAGCCTGGGACATCTAAGAACTTGG-3'

5'-ChlaTuA1_long969: 5'-GAAGAACCGCATGAACCCAGTCC-3'

3'-TuA6260: 5'-TTTAACAGGAAGCCGTCGGATAGC-3'

5'-Tua2-10g: 5'-GCCCCGATTGATTACGCTCAC-3'

3'-TuA2-3288g: 5'-GAATTACATTGTGTGGTTTTAACTGCAGGT-3'

5'-tub1-33c: 5'-GCTGCCAGGACCTTCATACG-3'

3'-tub1-1667c: 5'-TTGCGCCATTGCTATCACAC-3'

5'-EcoTuB2_upper: 5'-GTCACACGGCCCTCGGTACAC-3'

3'-XhoTuB2_lower: 5'-GCATCACCCTTGACACCAACAC-3'

5'-Tua1-1947c: 5'-CAAGTGTAGTGCTTTGCTG-3'

3'-Tua1-2290c: 5'-GTGCATGACTCCCAGT-3'

5'-Tua2-2403: 5'-CCGAGCTAGCATGTGAGCGTGTC-3'

3'-Tua2-2952c: 5'-ACGGGAGTGCAGGCGCTG-3'

5'-Tubs1818g: 5'-AACAACGTCAAGTCTTCCGTGTGC-3'

S2 Appendix. Procedures for supplemental experiments.

Northern hybridization

Tubulin mRNA expression in the disruptants was verified by northern hybridization analysis [1]. Total RNA was isolated using TRIzol Reagent (Thermo Fisher Scientific) in accordance with the manufacturer's instructions. To distinguish the mRNAs from the four tubulin genes, the 3'-UTR region of each tubulin gene was amplified from the wild-type genomic DNA using primers 5'-Tua1-1947c and 3'-Tua1-2290 (for *tua1*), 5'-Tua2-2403c and 3'-Tua2-2952c (for *tua2*), 5'-Tub1-3175c and 3'-Tub1-3683g (for *tub1*), and 5'-Tub2-3032c and 3'-Tub2-3507g (for *tub2*) together with PCR DIG Labeling Mix (Sigma-Aldrich), and the amplified 3'-UTR regions were used as probes.

Drug-resistance test by dilution series of the inoculum

C. reinhardtii strains were grown in liquid TAP medium until the mid-log phase, and then diluted or concentrated to dilution series, 5×10^6, 5×10^7, 5×10^8, and 5×10^9 cells/mL. Then, 3 µL of culture was spotted on a TAP-agar plate containing propyzamide (0–20 µM), oryzalin (0–20 µM), or colchicine (0–4 mM) and cultured for a week at 26°C under 12-h light/12-h dark conditions. A wild-type strain (CC-125) and an oryzalin-resistant mutant (upA12) [2], or colchicine-resistant mutants (*col^{R}4* and *col^{R}15* [3]) were used as references.

S3 Appendix. Supplementary references

1. Kato-Minoura T, Hirono M, Kamiya R. *Chlamydomonas* inner-arm dynein mutant, *ida5*, has a mutation in an actin-encoding gene. J Cell Biol. 1997;137(3):649–56.
2. James SW, Silflow CD, Stroom P, Lefebvre PA. A mutation in the a1-tubulin gene of *Chlamydomonas reinhardtii* confers resistance to anti-microtubule herbicides. J Cell Sci. 1993:209–18.
3. Lee VD, Huang B. Missense mutations at lysine 350 in beta 2-tubulin confer altered sensitivity to microtubule inhibitors in *Chlamydomonas*. Plant Cell. 1990;2(11):1051–7. Epub 1990/11/01. doi: 10.1105/tpc.2.11.1051. PubMed PMID: 2152107; PubMed Central PMCID: PMCPMC159953.
4. Ma C, Li C, Ganesan L, Oak J, Tsai S, Sept D, et al. Mutations in alpha-tubulin confer dinitroaniline resistance at a cost to microtubule function. Mol Biol Cell. 2007;18(12):4711–20. Epub 2007/09/21. doi: 10.1091/mbc.e07-04-0379. PubMed PMID: 17881728; PubMed Central PMCID: PMCPMC2096588.
5. Lyons-Abbott S, Sackett DL, Wloga D, Gaertig J, Morgan RE, Werbovetz KA, et al. alpha-Tubulin mutations alter oryzalin affinity and microtubule assembly properties to confer dinitroaniline resistance. Eukaryot Cell. 2010;9(12):1825–34. Epub 2010/09/28. doi: 10.1128/EC.00140-10. PubMed PMID: 20870876; PubMed Central PMCID: PMCPMC3008275.
6. Ryan LA, Hoey E, Trudgett A, Fairweather I, Fuchs M, Robinson MW, et al. *Fasciola hepatica* expresses multiple alpha- and beta-tubulin isotypes. Mol Biochem Parasitol. 2008;159(1):73–8. Epub 2008/03/29. doi: 10.1016/j.molbiopara.2008.02.001. PubMed PMID: 18372053; PubMed Central PMCID: PMCPMC3820024.
7. Morrissette NS, Mitra A, Sept D, Sibley LD. Dinitroanilines bind alpha-tubulin to disrupt microtubules. Mol Biol Cell. 2004;15(4):1960–8. Epub 2004/01/27. doi: 10.1091/mbc.e03-07-0530. PubMed PMID: 14742718; PubMed Central PMCID: PMCPMC379290.

8. Yin S, Zeng C, Hari M, Cabral F. Paclitaxel resistance by random mutagenesis of alpha-tubulin. Cytoskeleton (Hoboken). 2013;70(12):849–62. Epub 2013/10/25. doi: 10.1002/cm.21154. PubMed PMID: 24155014.

9. De S, Tsimounis A, Chen X, Rotenberg SA. Phosphorylation of alpha-tubulin by protein kinase C stimulates microtubule dynamics in human breast cells. Cytoskeleton (Hoboken). 2014;71(4):257–72. Epub 2014/02/28. doi: 10.1002/cm.21167. PubMed PMID: 24574051; PubMed Central PMCID: PMCPMC4113324.

10. Markovsky E, de Stanchina E, Itzkowitz A, Haimovitz-Friedman A, Rotenberg SA. Phosphorylation state of Ser(165) in alpha-tubulin is a toggle switch that controls proliferating human breast tumors. Cell Signal. 2018;52:74–82. Epub 2018/09/04. doi: 10.1016/j.cellsig.2018.08.021. PubMed PMID: 30176291; PubMed Central PMCID: PMCPMC6765385.

11. Poruchynsky MS, Kim JH, Nogales E, Annable T, Loganzo F, Greenberger LM, et al. Tumor cells resistant to a microtubule-depolymerizing hemiasterlin analogue, HTI-286, have mutations in alpha- or beta-tubulin and increased microtubule stability. Biochemistry. 2004;43(44):13944–54. Epub 2004/11/03. doi: 10.1021/bi049300+. PubMed PMID: 15518543.

12. Richards KL, Anders KR, Nogales E, Schwartz K, Downing KH, Botstein D. Structure-function relationships in yeast tubulins. Mol Biol Cell. 2000;11(5):1887–903. Epub 2000/05/04. doi: 10.1091/mbc.11.5.1887. PubMed PMID: 10793159; PubMed Central PMCID: PMCPMC14891.

13. Fromherz S, Giddings TH, Jr., Gomez-Ospina N, Dutcher SK. Mutations in alpha-tubulin promote basal body maturation and flagellar assembly in the absence of delta-tubulin. J Cell Sci. 2004;117(Pt 2):303–14. Epub 2003/12/17. doi: 10.1242/jcs.00859. PubMed PMID: 14676280.

14. Ishida T, Kaneko Y, Iwano M, Hashimoto T. Helical microtubule arrays in a collection of twisting tubulin mutants of *Arabidopsis thaliana*. Proc Natl Acad Sci USA. 2007;104(20):8544–9. Epub 2007/05/10. doi: 10.1073/pnas.0701224104. PubMed PMID: 17488810; PubMed Central PMCID: PMCPMC1895986.

15. Thitamadee S, Tuchihara K, Hashimoto T. Microtubule basis for left-handed helical growth in *Arabidopsis*. Nature. 2002;417(6885):193–6. Epub 2002/05/10. doi: 10.1038/417193a. PubMed PMID: 12000963.

16. Zou G, Ying SH, Shen ZC, Feng MG. Multi-sited mutations of beta-tubulin are involved in benzimidazole resistance and thermotolerance of fungal biocontrol agent *Beauveria bassiana*. Environ Microbiol. 2006;8(12):2096–105. Epub 2006/11/17. doi: 10.1111/j.1462-2920.2006.01086.x. PubMed PMID: 17107551.

17. Robinson MW, McFerran N, Trudgett A, Hoey L, Fairweather I. A possible model of benzimidazole binding to beta-tubulin disclosed by invoking an inter-domain movement. J Mol Graph Model. 2004;23(3):275–84. Epub 2004/11/09. doi: 10.1016/j.jmgm.2004.08.001. PubMed PMID: 15530823.

18. Ghisi M, Kaminsky R, Maser P. Phenotyping and genotyping of *Haemonchus contortus* isolates reveals a new putative candidate mutation for benzimidazole resistance in nematodes. Vet Parasitol. 2007;144(3-4):313–20. Epub 2006/11/15. doi: 10.1016/j.vetpar.2006.10.003. PubMed PMID: 17101226.

19. Park SY, Jung OJ, Chung YR, Lee CW. Isolation and characterization of a benomyl-resistant form of beta-tubulin-encoding gene from the phytopathogenic fungus *Botryotinia fuckeliana*. Mol Cells. 1997;7(1):104–9. Epub 1997/02/28. PubMed PMID: 9085273.

20. Ma Z, Yoshimura MA, Michailides TJ. Identification and characterization of benzimidazole resistance in *Monilinia fructicola* from stone fruit orchards in California. Appl Environ Microbiol. 2003;69(12):7145–52. Epub 2003/12/09. doi: 10.1128/aem.69.12.7145-7152.2003. PubMed PMID: 14660360; PubMed Central PMCID: PMCPMC309941.

21. Luo Y, Ma Z, Michailides TJ. Quantification of allele E198A in beta-tubulin conferring benzimidazole resistance in *Monilinia fructicola* using real-time PCR. Pest Manag Sci. 2007;63(12):1178–84. Epub 2007/10/04. doi: 10.1002/ps.1425. PubMed PMID: 17912681.

22. McKay GJ, Cooke LR. A PCR-based method to characterise and identify benzimidazole resistance in *Helminthosporium solani*. FEMS Microbiol Lett. 1997;152(2):371–8. Epub 1997/07/15. doi: 10.1111/j.1574-6968.1997.tb10454.x. PubMed PMID: 9231430.

23. Yang Y, Li MX, Duan YB, Li T, Shi YY, Zhao DL, et al. A new point mutation in beta2-tubulin confers resistance to carbendazim in *Fusarium asiaticum*. Pestic Biochem Physiol. 2018;145:15–21. Epub 2018/02/28. doi:

10.1016/j.pestbp.2017.12.006. PubMed PMID: 29482727.

24. Vela-Corcia D, Romero D, de Vicente A, Perez-Garcia A. Analysis of beta-tubulin-carbendazim interaction reveals that binding site for MBC fungicides does not include residues involved in fungicide resistance. Sci Rep. 2018;8(1):7161. Epub 2018/05/10. doi: 10.1038/s41598-018-25336-5. PubMed PMID: 29740047; PubMed Central PMCID: PMCPMC5940828.

25. Reijo RA, Cooper EM, Beagle GJ, Huffaker TC. Systematic mutational analysis of the yeast beta-tubulin gene. Mol Biol Cell. 1994;5(1):29–43. Epub 1994/01/01. doi: 10.1091/mbc.5.1.29. PubMed PMID: 8186463; PubMed Central PMCID: PMCPMC301007.

26. Jung MK, Wilder IB, Oakley BR. Amino acid alterations in the benA (beta-tubulin) gene of *Aspergillus nidulans* that confer benomyl resistance. Cell Motil Cytoskel. 1992;22(3):170–4. Epub 1992/01/01. doi: 10.1002/cm.970220304. PubMed PMID: 1423663.

27. Ziogas BN, Nikou D, Markoglou AN, Malandrakis AA, Vontas J. Identification of a novel point mutation in the β-tubulin gene of *Botrytis cinerea* and detection of benzimidazole resistance by a diagnostic PCR-RFLP assay. European journal of plant pathology. 2009;125(1):97–107.

28. Fujimura M, Oeda K, Inoue H, Kato T. A single amino-acid substitution in the beta-tubulin gene of *Neurospora* confers both carbendazim resistance and diethofencarb sensitivity. Curr Genet. 1992;21(4-5):399–404. Epub 1992/04/01. doi: 10.1007/bf00351701. PubMed PMID: 1388107.

29. Wiesen KM, Xia S, Yang CP, Horwitz SB. Wild-type class I beta-tubulin sensitizes taxol-resistant breast adenocarcinoma cells harboring a beta-tubulin mutation. Cancer Lett. 2007;257(2):227–35. Epub 2007/09/18. doi: 10.1016/j.canlet.2007.07.022. PubMed PMID: 17869412.

30. Cai M, Lin D, Chen L, Bi Y, Xiao L, Liu XL. M233I mutation in the beta-tubulin of *Botrytis cinerea* confers resistance to zoxamide. Sci Rep. 2015;5:16881. Epub 2015/11/26. doi: 10.1038/srep16881. PubMed PMID: 26596626; PubMed Central PMCID: PMCPMC4657022.

31. Wright AJ, Hunter CP. Mutations in a beta-tubulin disrupt spindle orientation and microtubule dynamics in the early *Caenorhabditis elegans* embryo. Mol Biol Cell. 2003;14(11):4512–25. Epub 2003/08/26. doi:

10.1091/mbc.e03-01-0017. PubMed PMID: 12937270; PubMed Central PMCID: PMCPMC268303.

32. Buhr TL, Dickman MB. Isolation, characterization, and expression of a second beta-tubulin-encoding gene from *Colletotrichum gloeosporioides* f. sp. aeschynomene. Appl Environ Microbiol. 1994;60(11):4155–9. Epub 1994/11/01. PubMed PMID: 7993097; PubMed Central PMCID: PMCPMC201950.

33. Fujimura M, Kamakura T, Inoue H, Yamaguchi I. Amino-acid alterations in the beta-tubulin gene of *Neurospora crassa* that confer resistance to carbendazim and diethofencarb. Curr Genet. 1994;25(5):418–22. Epub 1994/05/01. doi: 10.1007/bf00351780. PubMed PMID: 8082187.

34. Chen CJ, Yu JJ, Bi CW, Zhang YN, Xu JQ, Wang JX, et al. Mutations in a beta-tubulin confer resistance of *Gibberella zeae* to benzimidazole fungicides. Phytopathology. 2009;99(12):1403–11. Epub 2009/11/11. doi: 10.1094/PHYTO-99-12-1403. PubMed PMID: 19900007.

35. Liu X, Yin Y, Wu J, Jiang J, Ma Z. Identification and characterization of carbendazim-resistant isolates of *Gibberella zeae*. Plant Dis. 2010;94(9):1137–42. Epub 2010/09/01. doi: 10.1094/PDIS-94-9-1137. PubMed PMID: 30743730.

36. Escuin D, Burke PA, McMahon-Tobin G, Hembrough T, Wang Y, Alcaraz AA, et al. The hematopoietic-specific beta1-tubulin is naturally resistant to 2-methoxyestradiol and protects patients from drug-induced myelosuppression. Cell Cycle. 2009;8(23):3914–24. Epub 2009/11/11. doi: 10.4161/cc.8.23.10105. PubMed PMID: 19901556.

37. Schibler MJ, Huang B. The *colR4* and *colR15* beta-tubulin mutations in *Chlamydomonas reinhardtii* confer altered sensitivities to microtubule inhibitors and herbicides by enhancing microtubule stability. J Cell Biol. 1991;113(3):605–14. Epub 1991/05/01. doi: 10.1083/jcb.113.3.605. PubMed PMID: 1673126; PubMed Central PMCID: PMCPMC2288959.

38. Gaertig J, Thatcher TH, Gu L, Gorovsky MA. Electroporation-mediated replacement of a positively and negatively selectable beta-tubulin gene in *Tetrahymena thermophila*. Proc Natl Acad Sci USA. 1994;91(10):4549–53. Epub 1994/05/10. doi: 10.1073/pnas.91.10.4549. PubMed PMID: 7910408; PubMed Central PMCID: PMCPMC43823.

39. Gaertig J, Gao Y, Tishgarten T, Clark TG, Dickerson HW. Surface display

of a parasite antigen in the ciliate *Tetrahymena thermophila*. Nat Biotechnol. 1999;17(5):462–5. Epub 1999/05/20. doi: 10.1038/8638. PubMed PMID: 10331805.

40. Smith JJ, Yakisich JS, Kapler GM, Cole ES, Romero DP. A beta-tubulin mutation selectively uncouples nuclear division and cytokinesis in *Tetrahymena thermophila*. Eukaryot Cell. 2004;3(5):1217–26. Epub 2004/10/08. doi: 10.1128/EC.3.5.1217-1226.2004. PubMed PMID: 15470250; PubMed Central PMCID: PMCPMC522614.

41. Hari M, Wang Y, Veeraraghavan S, Cabral F. Mutations in alpha- and beta-tubulin that stabilize microtubules and confer resistance to colcemid and vinblastine. Mol Cancer Ther. 2003;2(7):597–605. Epub 2003/07/29. PubMed PMID: 12883031.

42. Hua XH, Genini D, Gussio R, Tawatao R, Shih H, Kipps TJ, et al. Biochemical genetic analysis of indanocine resistance in human leukemia. Cancer Res. 2001;61(19):7248–54. Epub 2001/10/05. PubMed PMID: 11585762.

43. Gokmen-Polar Y, Escuin D, Walls CD, Soule SE, Wang Y, Sanders KL, et al. beta-tubulin mutations are associated with resistance to 2-methoxyestradiol in MDA-MB-435 cancer cells. Cancer Res. 2005;65(20):9406–14. Epub 2005/10/19. doi: 10.1158/0008-5472.CAN-05-0088. PubMed PMID: 16230404.

44. de Lourdes Mottier M, Prichard RK. Genetic analysis of a relationship between macrocyclic lactone and benzimidazole anthelmintic selection on Haemonchus contortus. Pharmacogenet Genomics. 2008;18(2):129–40. Epub 2008/01/15. doi: 10.1097/FPC.0b013e3282f4711d. PubMed PMID: 18192899.

45. Ruiz F, Dupuis-Williams P, Klotz C, Forquignon F, Bergdoll M, Beisson J, et al. Genetic evidence for interaction between eta- and beta-tubulins. Eukaryot Cell. 2004;3(1):212–20. Epub 2004/02/12. doi: 10.1128/ec.3.1.212-220.2004. PubMed PMID: 14871951; PubMed Central PMCID: PMCPMC329518.

46. Jung MK, Oakley BR. Identification of an amino acid substitution in the benA, beta-tubulin gene of *Aspergillus nidulans* that confers thiabendazole resistance and benomyl supersensitivity. Cell Motil Cytoskel. 1990;17(2):87–94. Epub 1990/01/01. doi: 10.1002/cm.970170204. PubMed PMID: 2257633.

47. de Lourdes Mottier M, Prichard RK. Genetic analysis of a relationship between macrocyclic lactone and benzimidazole anthelmintic selection on

Haemonchus contortus. Pharmacogenetics and genomics. 2008;18(2):129–40.
