# Supplementary figures and images for: Chlamydomonas reinhardtii tubulin-gene disruptants for efficient isolation of strains bearing tubulin mutations
Source: PLoS One. 2020 Nov 23;15(11):e0242694. doi: 10.1371/journal.pone.0242694 (PMC7682851; doi:10.1371/journal.pone.0242694)

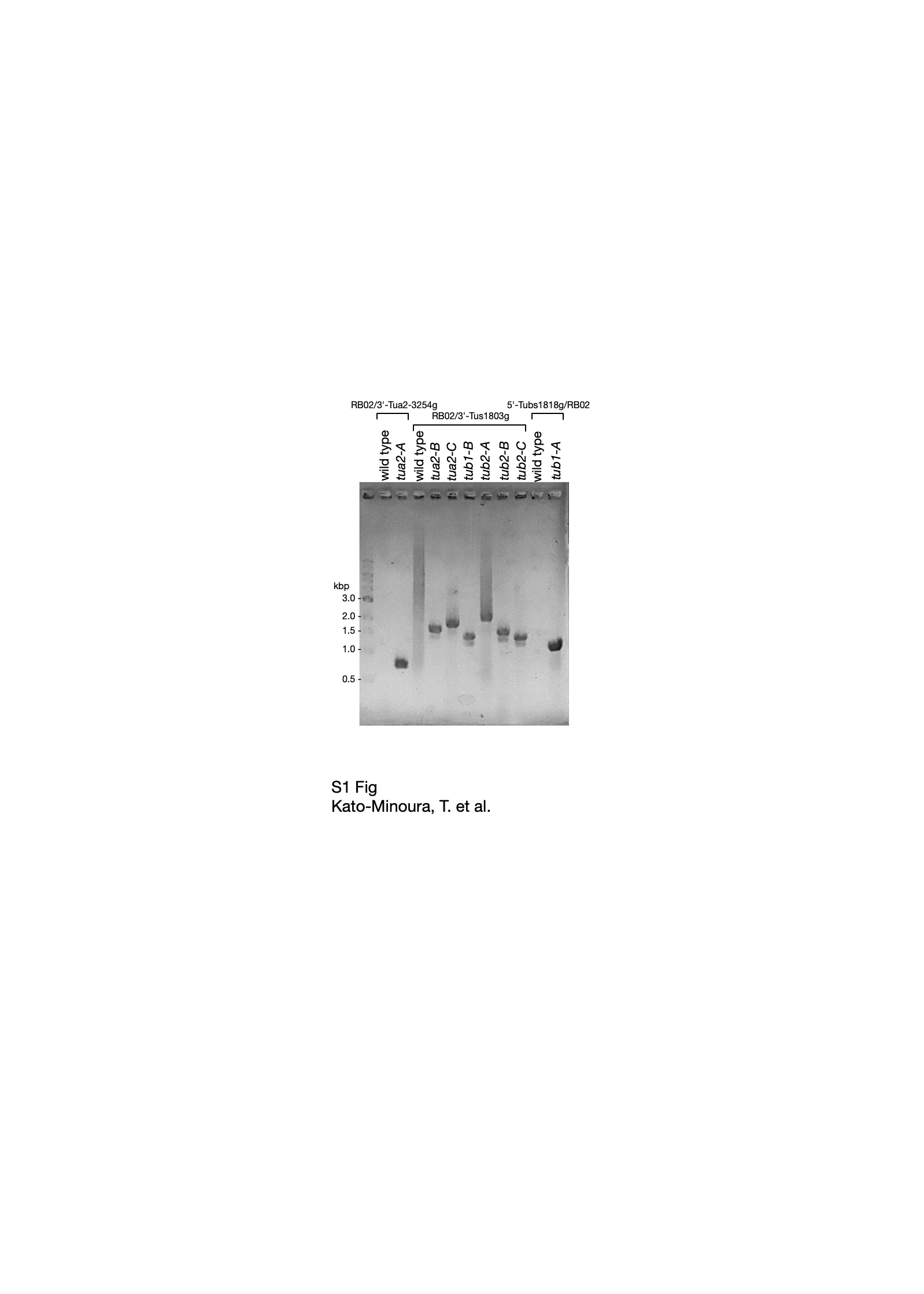

Supplement: S1 Fig — In each disruptant, the structure of the affected tubulin gene was confirmed by PCR using primers specific to AphVIII (RB02) and the target tubulin gene. A wild type (CC-125) genomic DNA was used as the template for negative controls. PCR-amplified gene fragments contained the inserted AphVIII cassette from the genomic DNA templates extracted from the disruptants but not the wild type. The sizes of the amplified fragments matched those predicted from the manner of AphVIII gene insertion (Fig 1), which was determined by sequencing in the vicinity of their disrupted tubulin gene. (TIF) [file pone.0242694.s002.tif]

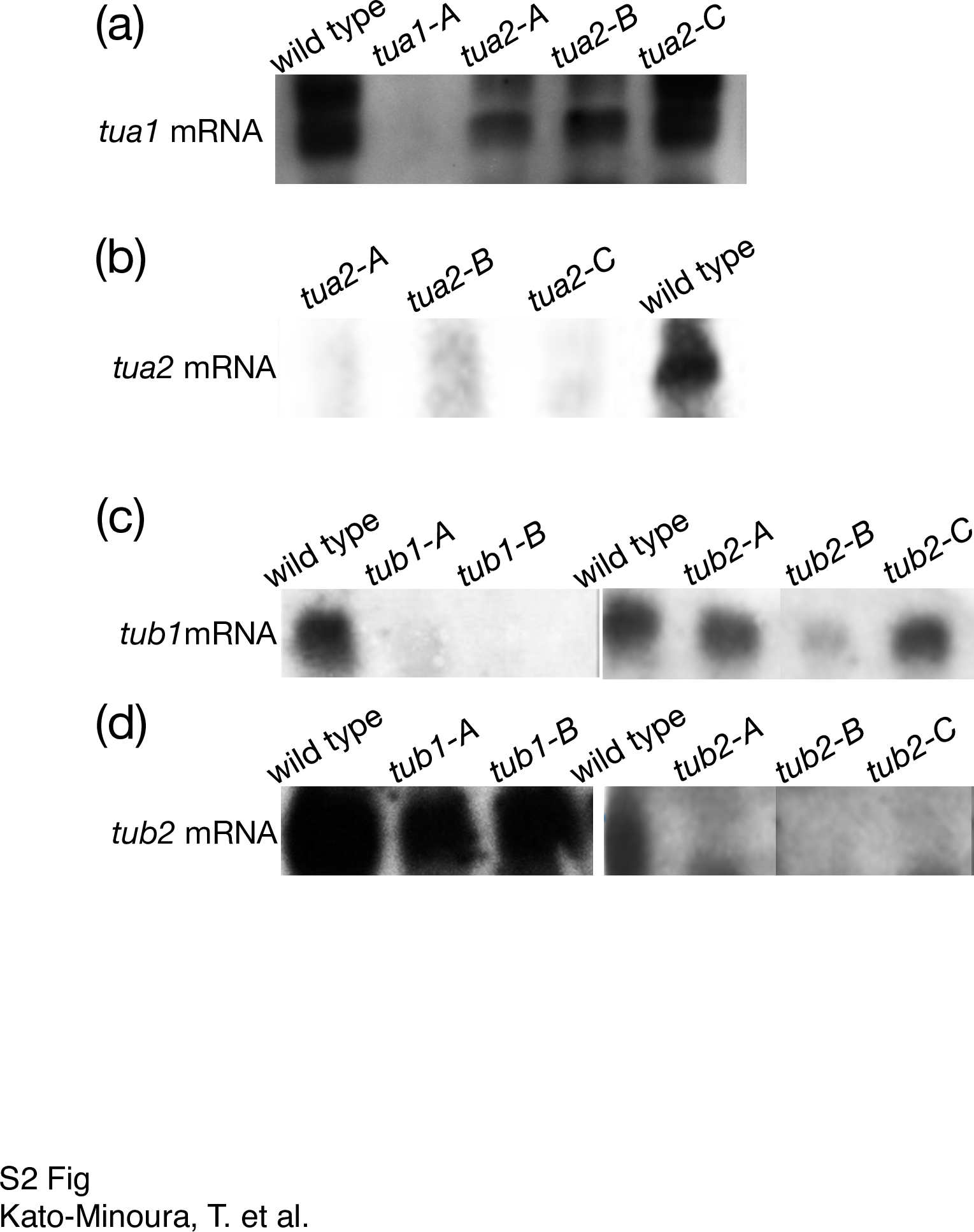

Supplement: S2 Fig — (a) tua1 transcripts. Total RNA from a wild type (CC-125), tua1-A, tua2-A, tua2-B, and tua2-C were hybridized with tua1 mRNA-specific probes. (b) tua2 transcripts. Total RNA from tua2-A, tua2-B, tua2-C, and wild type were hybridized with tua2 mRNA-specific probes. (c) tub1 transcripts. Total RNA from wild type, tub1-A, tub1-B, tub2-A, tub2-B, and tub2-C were hybridized with tub1 mRNA-specific probes. (d) tub2 transcripts. Total RNA from wild type, tub1-A, tub1-B, tub2-A, tub2-B, and tub2-C were hybridized with tub2 mRNA-specific probes. (TIF) [file pone.0242694.s003.tif]

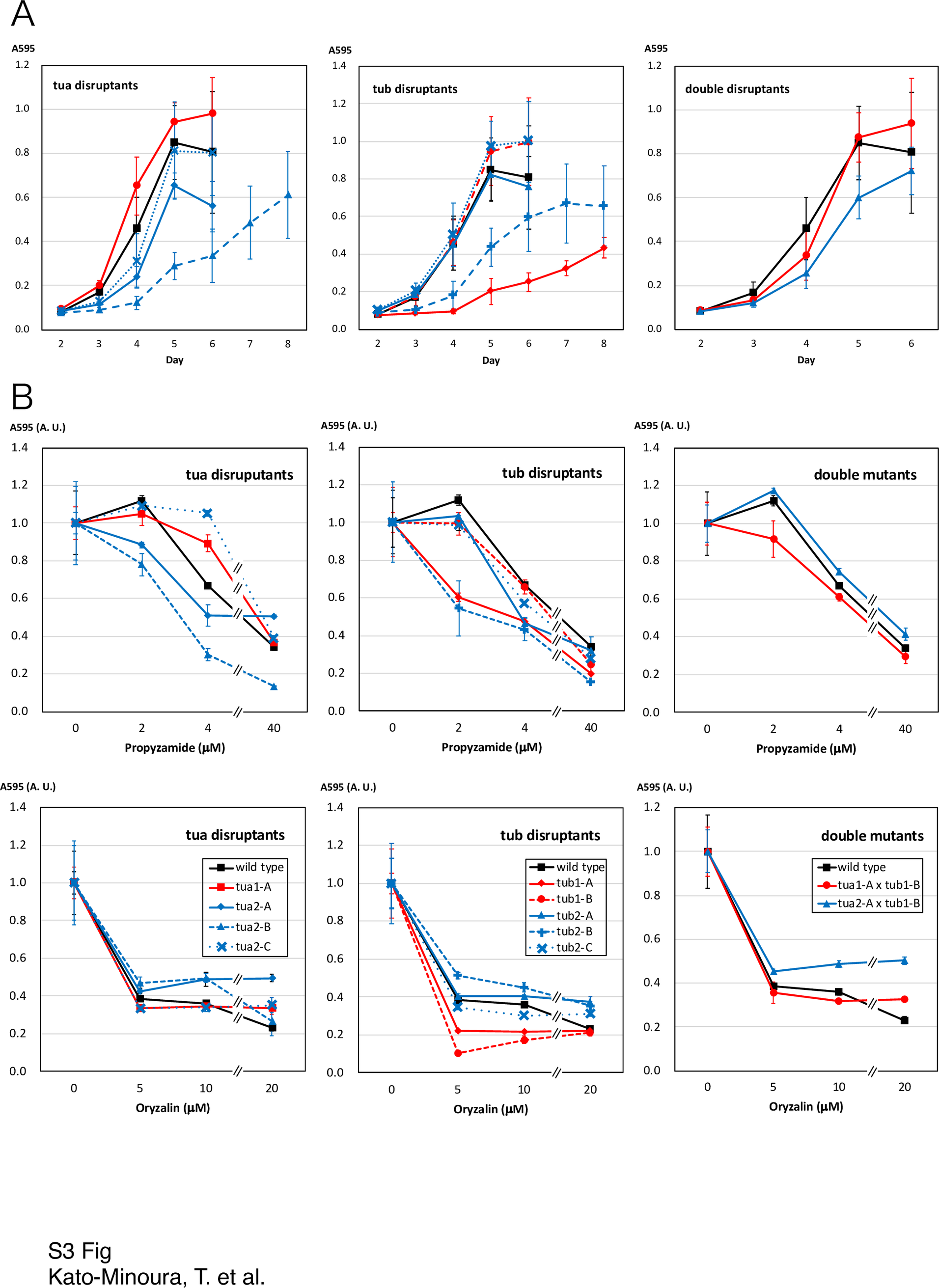

Supplement: S3 Fig — Cells were cultured in growth medium containing different concentrations of anti-tubulin drugs. Absorbance of the culture was measured at 595 nm every day. For comparison, data of a wild type (CC-124) was also collected. Error bars represent standard deviations of three different measurements. A: Growth rate of the tubulin disruptants. B: Drug sensitivities. Relative optical densities on day 5 are shown for all strains except for slow-growing strains, tua2-B, tub1-A, and tub2-B. For the slow-growing strains, data on day 8 are shown. In each case, the optical density observed without a drug is normalized to 1. (TIF) [file pone.0242694.s004.tif]

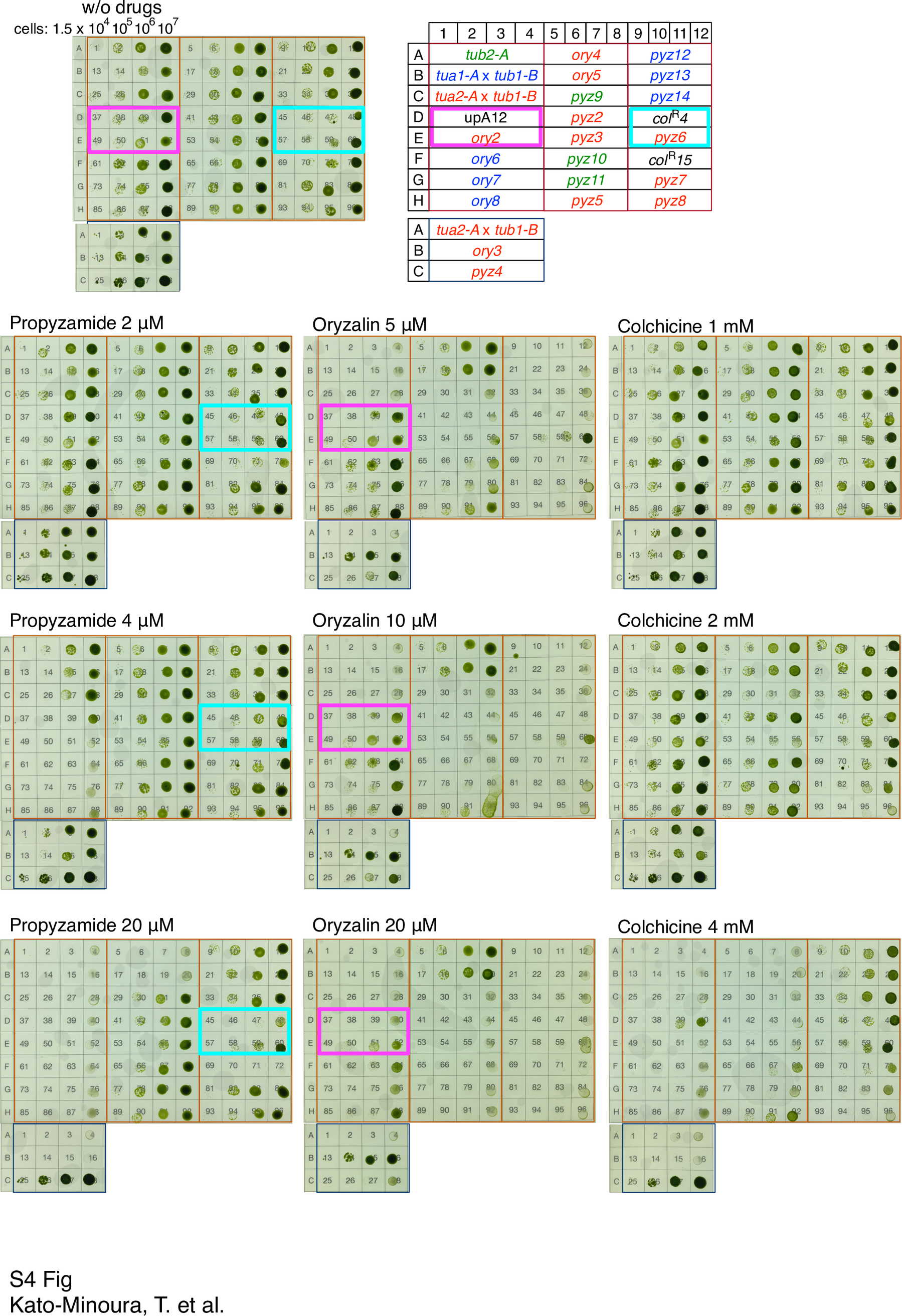

Supplement: S4 Fig — Serial dilutions of the cells were inoculated onto TAP/agar medium containing various concentrations of propyzamide, oryzalin and colchicine, and cultured for 7 days. The names of strains are given in the table (upper right corner) in different colors reflecting their parent strains. Three parent strains and three previously reported strains, upA12, colR4, and colR15, were also cultured and compared with ory2 and pyz6 having the same mutations (boxed). The latter single-tubulin-gene mutants show slightly stronger drug resistance as seen at high drug concentrations. (TIF) [file pone.0242694.s005.tif]

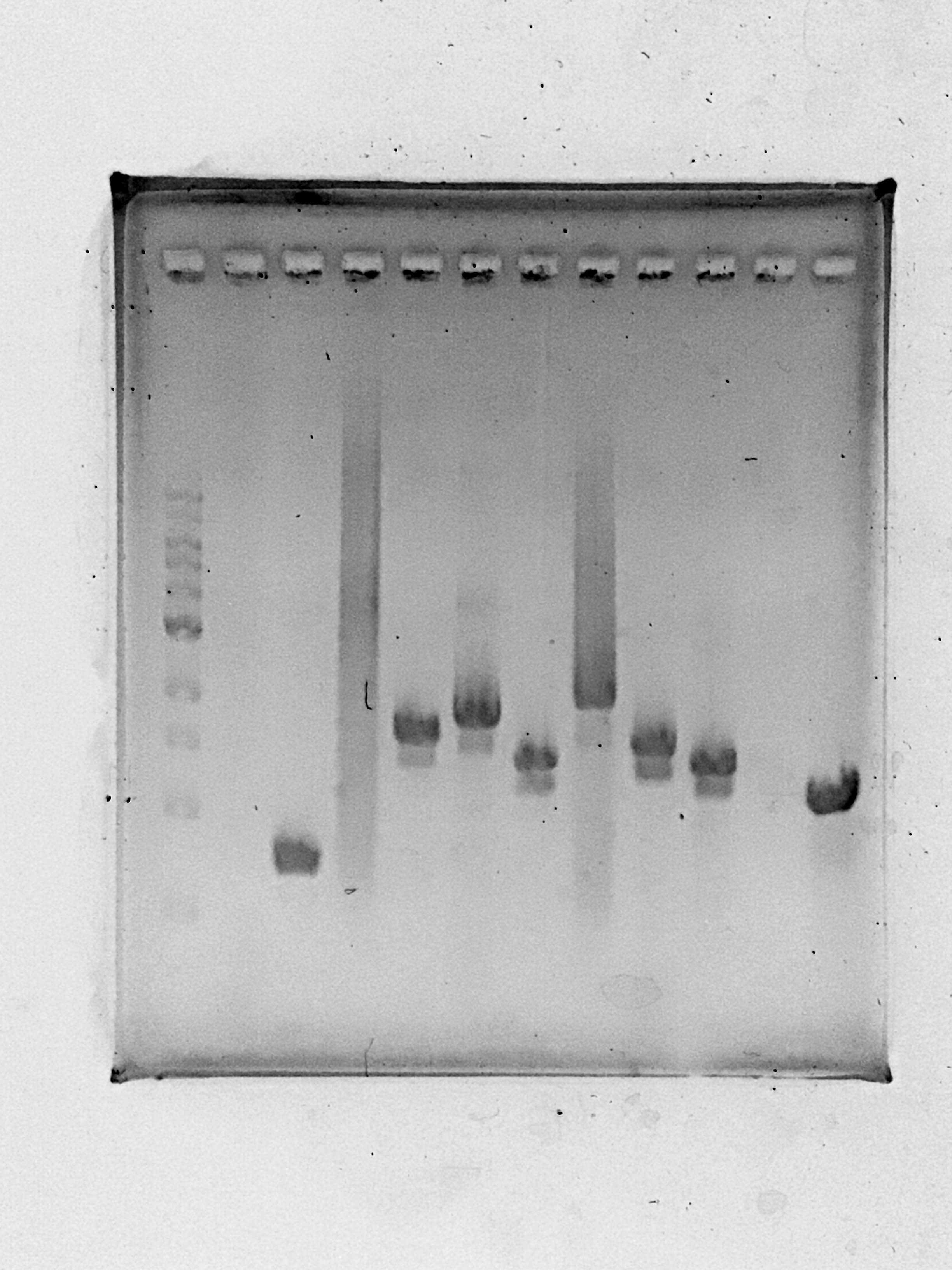

Supplement: S1 Raw images — (JPEG) [file pone.0242694.s009.jpeg]

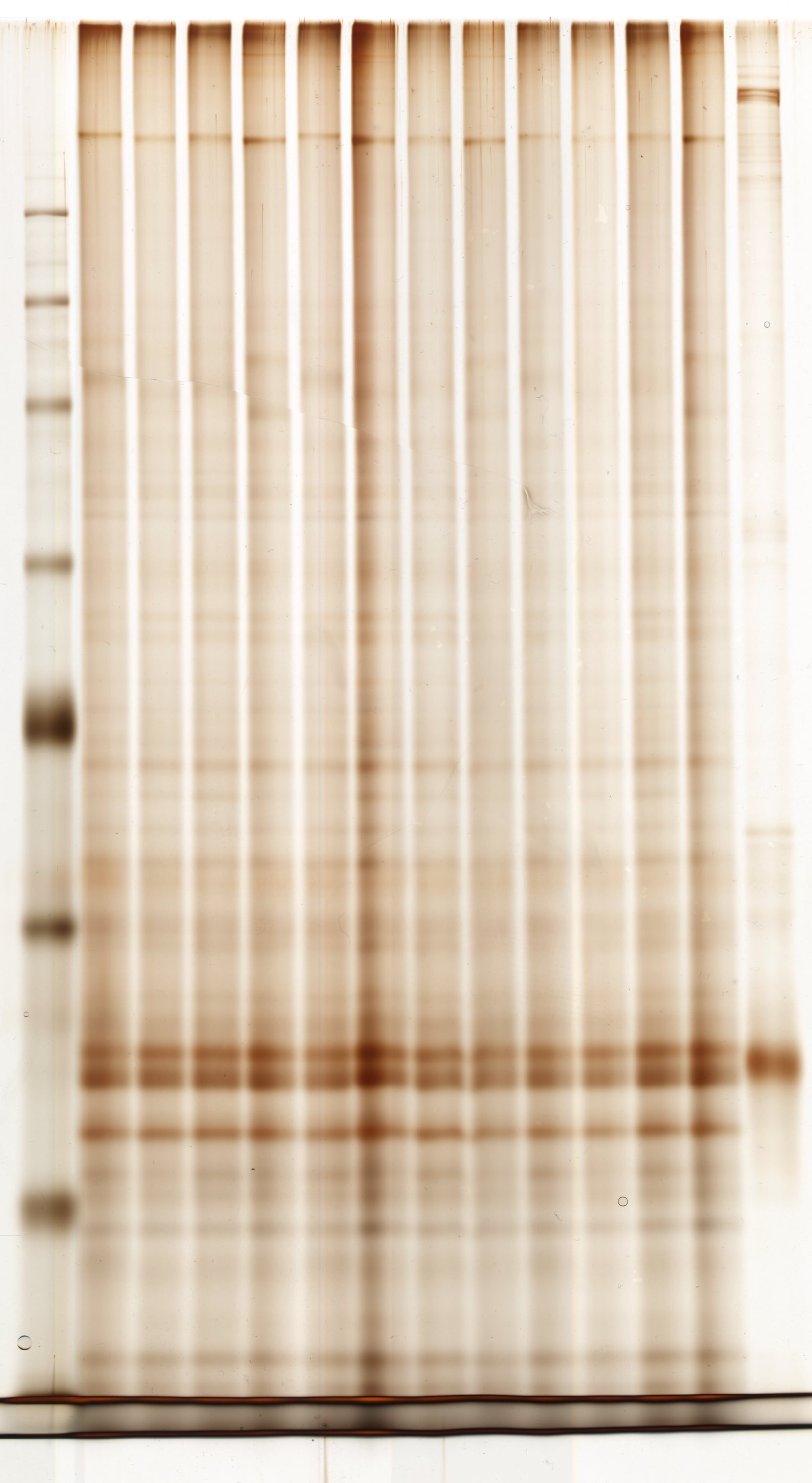

Supplement: S3 Raw images — (JPEG) [file pone.0242694.s011.jpeg]

wild type (x5)

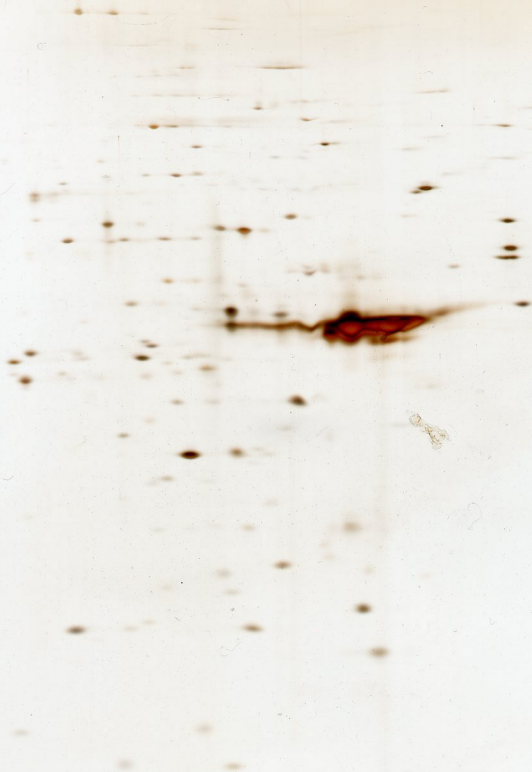

wild type

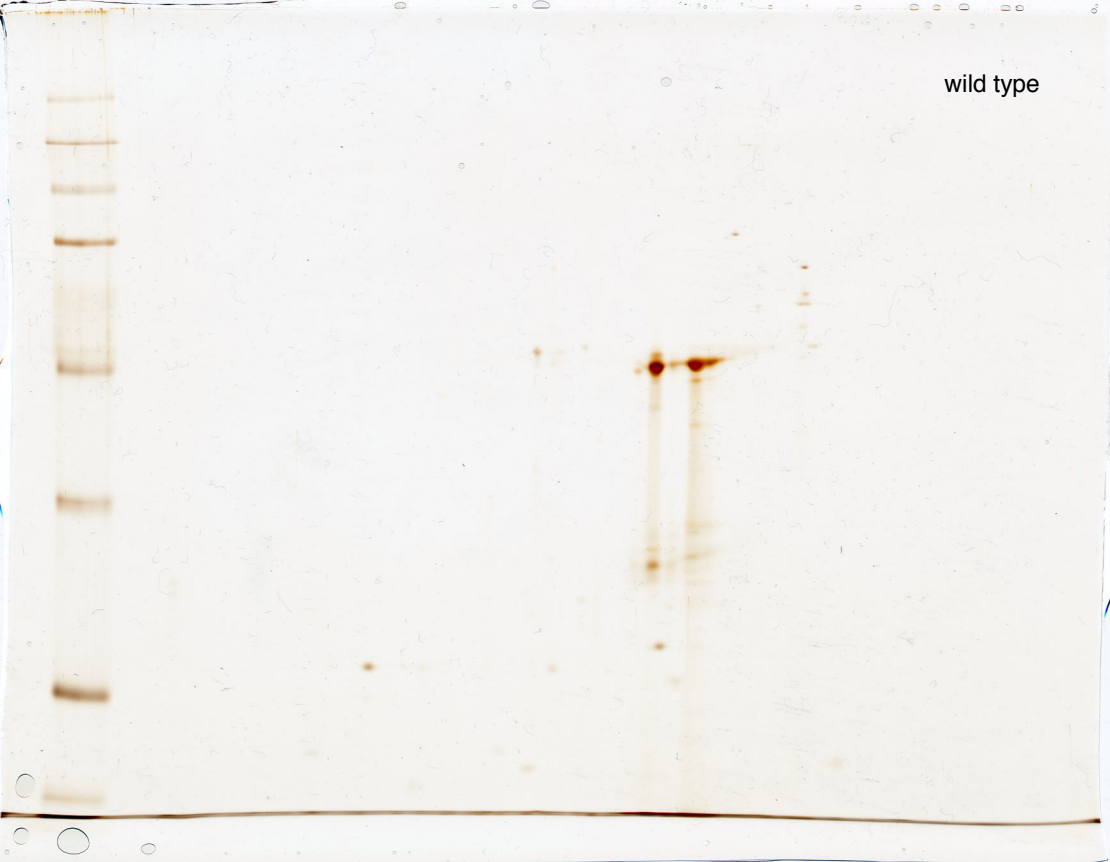

pyz2

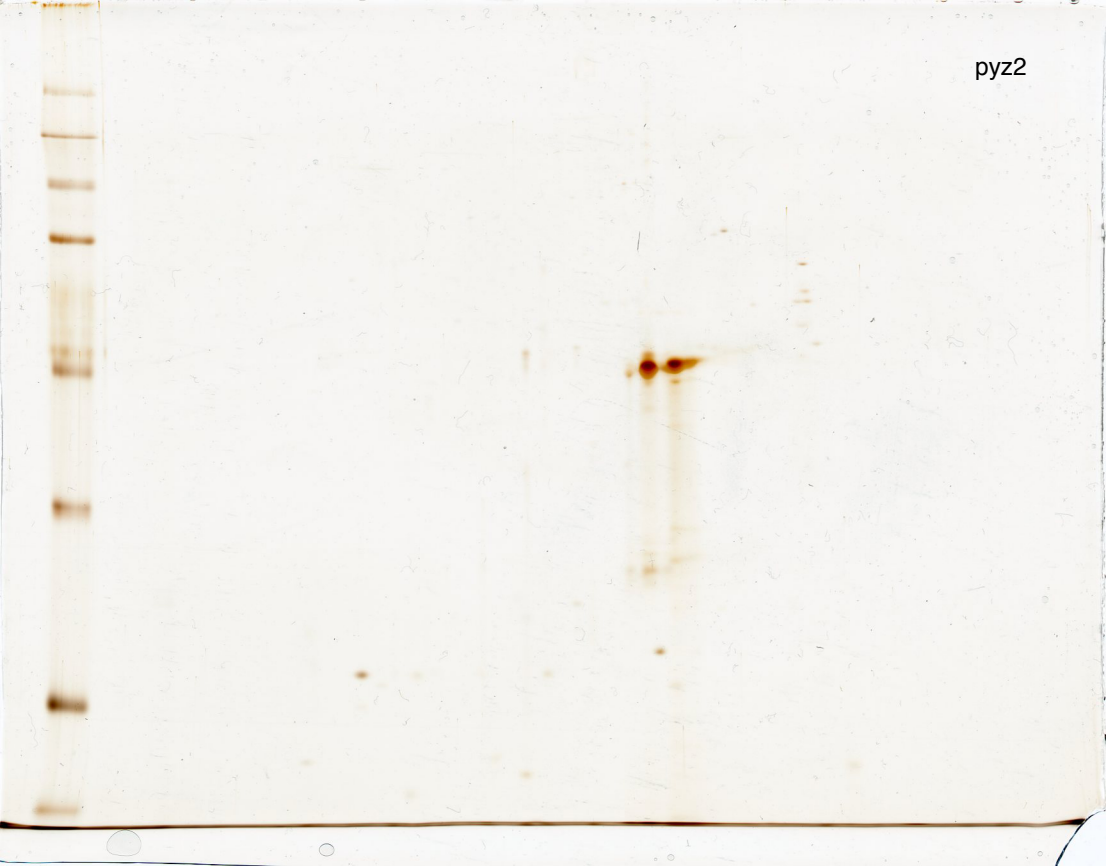

pyz10

Supplement: S4 Raw images — (PDF) [file pone.0242694.s012.pdf]
