# Supplementary material for: Chlamydomonas reinhardtii tubulin-gene disruptants for efficient isolation of strains bearing tubulin mutations
Source: PLoS One. 2020 Nov 23;15(11):e0242694. doi: 10.1371/journal.pone.0242694 (PMC7682851; doi:10.1371/journal.pone.0242694)
Supplement: S2 Raw images — (PDF) [file pone.0242694.s010.pdf]

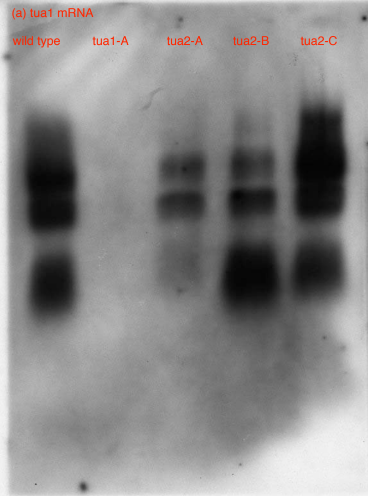
(a) tua1 mRNA
wild type
tua1-A
tua2-A
tua2-B
tua2-C

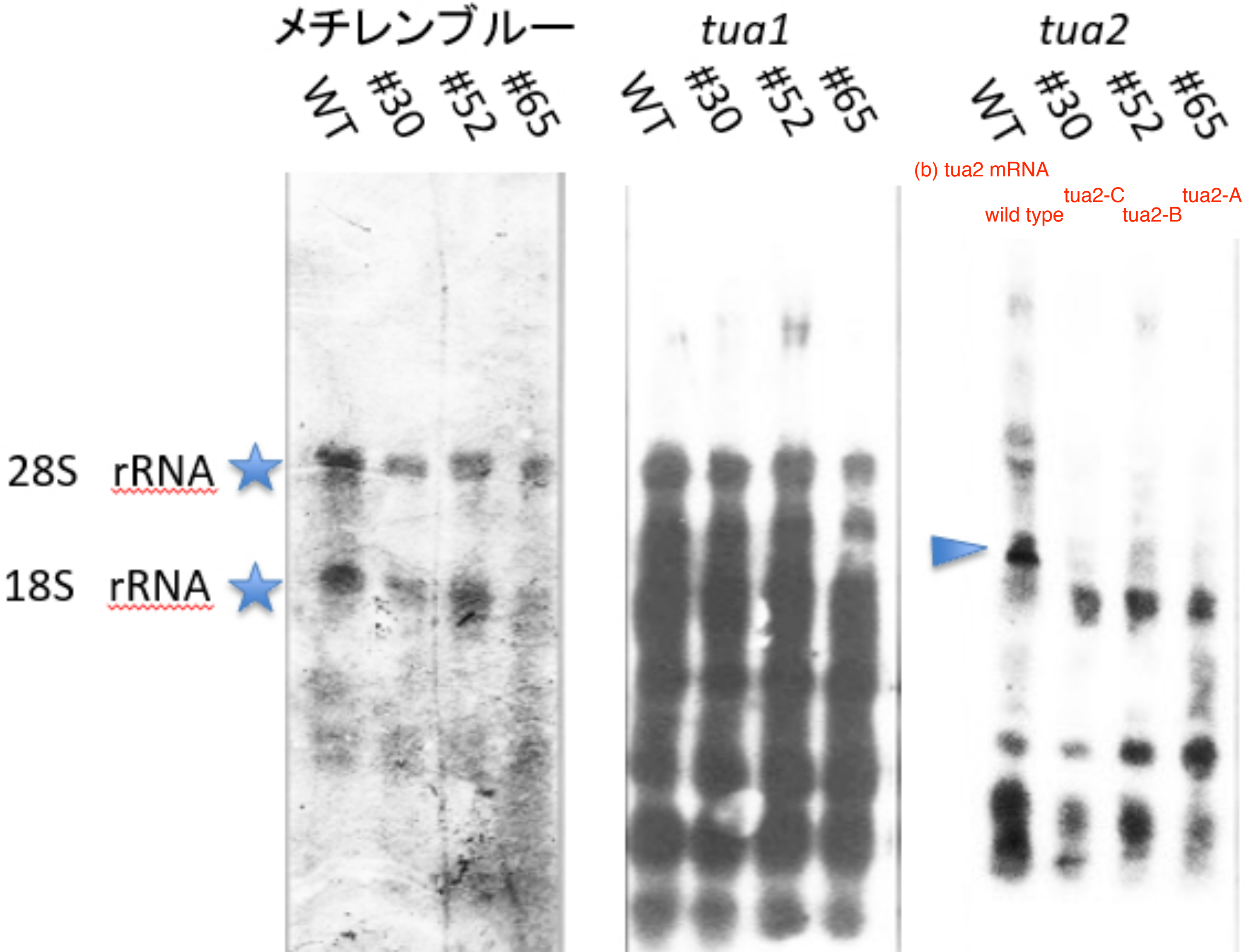
メチレンブルー
WT #30 #52 #65
tua1
WT #30 #52 #65
tua2
WT #30 #52 #65
(b) tua2 mRNA
wild type
tua2-C
tua2-B
tua2-A
28S rRNA
18S rRNA

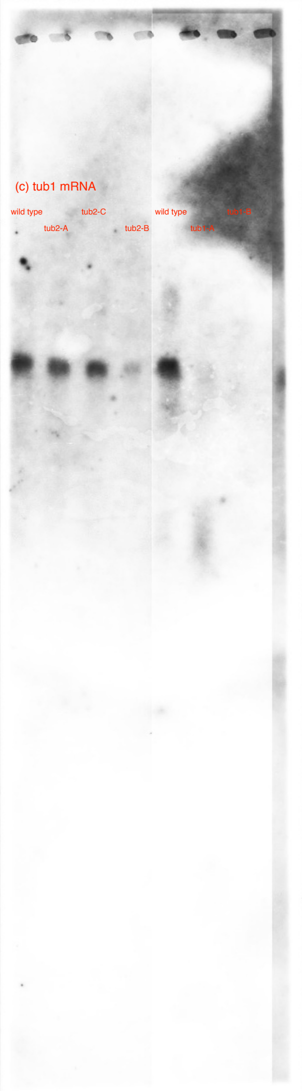
(c) tub1 mRNA
wild type
tub2-A
tub2-C
tub2-B
wild type
tub1-A
tub1-B

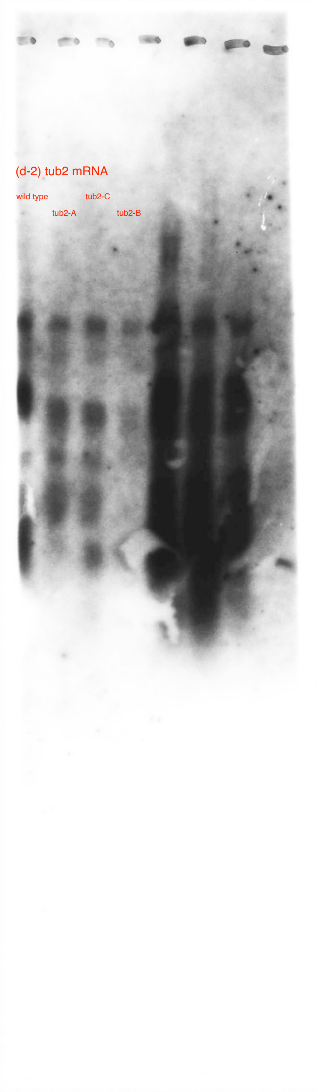
(d-2) tub2 mRNA
wild type
tub2-A
tub2-C
tub2-B

③ B1

④ B2

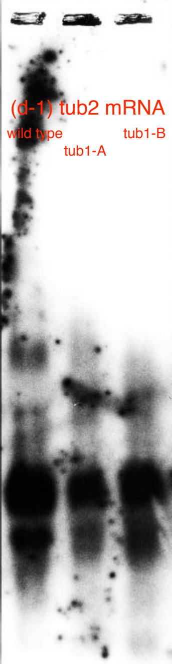
(d-1) tub2 mRNA
wild type
tub1-A
tub1-B
